# An Updated Mortality and Community Discharge Prognostic Model for Older Adults Admitted to Skilled Nursing Facilities for Post-Acute Care

**DOI:** 10.1016/j.jamda.2025.106103

**Published:** 2026-02-12

**Authors:** W. James Deardorff, Siqi Gan, Bocheng Jing, Kenneth Lam, W. John Boscardin, Alexander K. Smith, Sei J. Lee

**Affiliations:** a Division of Geriatrics, University of California, San Francisco, San Francisco, CA, USA; b Philip R. Lee Institute for Health Policy Studies, University of California, San Francisco, San Francisco, CA, USA; c Northern California Institute for Research and Education, San Francisco, CA, USA; d Department of Epidemiology and Population Health, Stanford University, Palo Alto, CA, USA; e Division of Geriatric Medicine, Department of Medicine, University of Colorado School of Medicine, Aurora, CO, USA; f Department of Epidemiology and Biostatistics, University of California, San Francisco, San Francisco, CA, USA; g Geriatrics, Palliative and Extended Care Service Line, San Francisco Veterans Affairs Health Care System, San Francisco, CA, USA

**Keywords:** Prognostic model, skilled nursing facility, post-acute care, mortality, Minimum Data Set

## Abstract

**Objectives:**

We previously developed a multi-outcome prognostic model for older adults admitted to skilled nursing facilities (SNFs) for short-term rehab using Medicare data. However, incorporating predictors from the Minimum Data Set (MDS), a mandated comprehensive assessment, may improve model performance. This study sought to develop an updated model with MDS elements for use on day 7 of SNF admission when clinical trajectories are more established.

**Design:**

Retrospective cohort study.

**Setting and Participants:**

Twenty percent national sample of community-dwelling Medicare Fee-for-Service beneficiaries aged ≥66 admitted to an SNF for at least 7 days following a hospitalization between 2017 and 2019.

**Methods:**

We predicted 2 outcomes: 6-month mortality and successful community discharge (community discharge without rehospitalization or death in the subsequent 30 days). For model development, we started with predictors from our published Medicare-based model (age, sex, Medicaid status, discharge diagnosis, hospital length of stay, admission type, comorbidities, prior hospitalizations), used Least Absolute Shrinkage and Selection Operator (LASSO) on MDS elements for variable selection, and performed logistic regression to determine predictor coefficients. Model performance was assessed by concordance statistics (c-statistics), calibration plots, and decision curve analysis.

**Results:**

The cohort included 426,680 individuals [mean age 81.3 years (SD = 8.3), 62.7% female, 7.9% Black]. Overall, 19.9% died within 6 months, and 57.6% experienced a successful community discharge. The updated MDS model, which included Medicare predictors and 6 MDS items (activities of daily living score, cognitive status, urinary incontinence, bowel incontinence, oxygen use, walking balance), showed improvements over the Medicare model in discrimination [bootstrapped optimism-corrected c-statistic of 0.789 (95% CI, 0.787–0.790) vs 0.747 (95% CI, 0.745–0.749) for 6-month mortality and 0.730 (95% CI, 0.728–0.731) vs 0.685 (95% CI, 0.683–0.687) for successful community discharge, respectively], net benefit, and fraction of new information. Models showed good calibration.

**Conclusions and Implications:**

Incorporating MDS data from the first 7 days of SNF admission improved the accuracy of predictions of 6-month mortality and successful community discharge.

Roughly 20% of hospitalized older adults are discharged from a hospital to a skilled nursing facility (SNF) for short-term post-acute care.^1^ SNFs provide skilled services, such as nursing care, physical therapy, and occupational therapy, with the goal of helping patients rehabilitate after an acute illness or surgery before returning home. However, the post-acute care period at an SNF is a particularly vulnerable time for older adults, with ~20% dying within 6 months and only 50% experiencing a successful discharge home.^1–4^ Many older adults, particularly those with serious illness and frailty, cycle between SNFs and hospitals in a period of declining health and function before death, termed by others as being “rehabbed to death.”^5,6^ To frame goals-of-care conversations and manage expectations around functional recovery, prognostic models play an essential role in providing clinical care at an SNF that aligns with the values and preferences of patients and caregivers.

We previously reported the development of a clinical prediction model for older adults receiving short-term care at an SNF that is available for SNF clinicians to use through the ePrognosis website, an online compendium of prognostic models for older adults.^2,7^ This model provides risk estimates for 6-month mortality and successful community discharge and demonstrated good discrimination and calibration. Although this model could be used directly on SNF admission, an updated model that provides risk estimates on day 7 of SNF admission may be more clinically useful to SNF clinicians to guide goals-of-care discussions and discharge planning, given that an individual’s health trajectory may be much clearer at this time. In addition, this updated model can incorporate data elements from the Minimum Data Set (MDS), a mandated assessment that is typically performed within a few days of SNF admission.^8,9^ The MDS provides comprehensive measures on prognostically important predictors not well captured in Medicare hospitalization data, including an individual’s functional status [eg, impairments in activities of daily living (ADLs)], cognition, delirium status, behavioral issues, pressure injuries, falls, and specific therapies/medications (eg, oxygen use, antipsychotics, diuretics).

Therefore, the aim of the current study was to develop an updated prognostic model for 6-month mortality and community discharge using MDS data elements (available 7 days into SNF stay) and compare its performance with a Medicare data–only base model.

## Methods

### Design

This was a prognostic modeling study using a retrospective cohort design to develop and internally validate an updated multivariable prediction model. We followed the 2024 Transparent Reporting of a multivariable prediction model for Individual Prognosis Or Diagnosis + Artificial Intelligence (TRIPOD + AI) statement to report this study (Supplementary Table 1).^10^

### Sample

Using data from a 20% national sample of Medicare Fee-for-Service beneficiaries, we created a cohort of community-dwelling older adults aged ≥66 discharged to an SNF following a hospitalization between 2017 and 2019 (Supplementary Methods). We excluded individuals aged <66 years, those who did not have continuous Medicare Fee-for-Service coverage during the post-acute period, individuals residing in a nursing home in the 3 months before SNF admission, and individuals who were not in the community directly before the index hospitalization.

We further refined our cohort based on a primary goal to incorporate MDS data elements (Supplementary Methods). Because many MDS assessments are not completed for several days following SNF admission and use a 7-day lookback period, we included individuals who remained at an SNF on day 7 and had a comprehensive admission MDS assessment on or before day 7. Therefore, we excluded individuals who were either discharged, re-admitted to the hospital, or died soon after SNF admission. This cohort reflects individuals who remain at an SNF for a longer period and may most benefit from prognostic discussions.

### Procedure

We predicted 2 outcomes: 6-month all-cause mortality and “successful community discharge.” Information on 6-month all-cause mortality was collected from the Master Beneficiary Summary File and the National Death Index, which displays high accuracy in identifying deaths.^11^ Successful community discharge was defined based on the Centers for Medicare and Medicaid Services (CMS) quality measure as a community discharge from SNFs with no rehospitalization or death in the 30 days following discharge.^4,12,13^

Our previously published prognostic model included the predictors of age, sex, Medicaid enrollment, type of admission (eg, urgent/emergent vs elective), hospital length of stay, principal hospital discharge diagnosis, surgical procedures, number of hospitalizations in the past year, and chronic conditions (Supplementary Table 2).^2^ After feedback from clinicians regarding the difficulty of ascertaining discharge diagnosis categories, we simplified the categories to include injury (eg, fractures), diseases of the circulatory system (eg, heart failure, stroke), musculoskeletal conditions (eg, knee and hip replacement), neoplasms (eg, admissions for chemotherapy), and all other diagnoses (Supplementary Table 3). We also removed the surgical procedures variable, as it may be difficult for clinicians to ascertain, and performance was similar without it.

We considered several predictors from the admission MDS assessment to include in the updated MDS model based on previous studies (Supplementary Table 4).^14–19^ These included cognitive status (based on the Cognitive Function Scale), functional status (based on the MDS-ADL long-form scale with scores ranging from 0 to 28 based on performance on 7 ADLs including bed mobility, transfers, locomotion on unit, dressing, eating, toilet use, and personal hygiene), balance while walking, delirium (based on the Confusion Assessment Method), behavioral issues (based on the Agitated and Reactive Behavior Scale), urinary and bowel incontinence, shortness of breath, pressure ulcers, oxygen use, and medication use (eg, antipsychotics, diuretics).^20–23^

### Data Analysis

To develop the base Medicare model, we performed logistic regression using the prespecified Medicare-derived predictors as above. To develop the updated MDS model, we first performed variable selection only on the MDS predictors using Least Absolute Shrinkage and Selection Operator (LASSO), forcing the Medicare-derived predictors into the model. We performed multiple imputation to account for the small amount of missing data with MDS elements (generally <5%) (Supplementary Methods). Given our goal to develop a parsimonious model to use on ePrognosis, the final updated MDS model included a limited number of MDS variables chosen by assessing the order in which variables were selected by LASSO across the 2 outcomes (Supplementary Methods).

We compared model performance between the base Medicare model and the updated MDS model by examining discrimination, calibration, Brier score, net benefit using decision curve analysis, and fraction of new information provided (Supplementary Methods).^24–27^ For discrimination, we calculated the concordance statistic (c-statistic) (ideal value of 1).^25^ For calibration, we show calibration plots and calculated the calibration slope (ideal value of 1), intercept (ideal value of 0), and integrated calibration index (ICI) or Eavg (weighted difference between observed and predicted probabilities over the range of predicted probabilities; ideal value of 0).^28^ We also calculated the Brier score as an overall measure of model performance.^25^ To quantify optimism in measures of model performance, we performed internal validation via bootstrapping with 100 samples, given methodologic guidance favoring this form of internal validation over other approaches.^24,29,30^ We conducted a decision curve analysis to compare the net benefit between models with and without MDS data elements, with age and sex alone, and against the “treat all” (“intervene for all”) and “treat none” (“intervene for none”) strategies (Supplementary Methods).^27,31^ Net benefit is a quantitative measure of a model’s clinical utility that takes into account expected benefits and harms for certain interventions (eg, comprehensive care planning, palliative care referrals, or medication reviews for individuals at high mortality risk may be helpful but are time- and resource-intensive). We examined net benefit across thresholds from 0% to 100% given that the model may inform a variety of interventions. We also calculated the fraction of new information provided, which refers to the proportion of total predictive information explained by MDS data elements when added to the base Medicare model.^32^

Finally, as a secondary analysis, we examined model performance across clinically relevant subgroups by age groups, sex, race/ethnicity, and hospital discharge diagnosis. Statistical analyses were performed using SAS version 9.4 (SAS Institute, Inc), Stata version 18 (StataCorp LLC), and R version 4.4.1 (R Project for Statistical Computing). The study was reviewed and approved by the University of California, San Francisco, Committee on Human Research.

## Results

### Cohort Characteristics

After applying exclusion criteria, the final cohort included 426,680 individuals who remained at an SNF on day 7 and had an MDS assessment performed (Supplementary Figure 1). The mean age was 81.3 years (standard deviation = 8.3), 267,389 (62.7%) were women, 33,502 (7.9%) were Black, 16,173 (3.8%) were Hispanic, and 362,591 (85.0%) were White (Table 1, Supplementary Table 5). The cohort had a high prevalence of chronic conditions such as heart failure (n = 154,437; 36.2%), complicated diabetes (n = 130,844; 30.7%), and dementia (n = 118,818; 27.8%). On the MDS-ADL score (which ranges from 0 to 28), the mean was 17.0 (standard deviation = 3.99), which indicates needing extensive assistance in most ADLs. On the Cognitive Function Scale, 89,581 (21.0%) had mild impairment and 61,315 (14.4%) had moderate impairment. A total of 84,714 individuals (19.9%) died within 6 months and 245,591 (57.6%) experienced a successful community discharge.

### Model Development

Supplementary Tables 6 and 7 show the unadjusted odds ratios for all predictors for the 2 outcomes. Table 2 displays the multivariable-adjusted odds ratios for variables in the final MDS model following LASSO variable selection. MDS elements included the MDS-ADL scale score, cognitive status, oxygen use, urinary incontinence, bowel incontinence, and balance while walking. Supplementary Tables 8 and 9 show the intercept and full model coefficients for the 2 outcomes. Supplementary Figures 2 and 3 show plots of the proportion of overall χ^2^ for each predictor for the 2 outcomes. Increasing age, certain comorbidities (eg, metastatic cancer, weight loss), discharge diagnosis, cognitive status, and oxygen use were most predictive of increased mortality. Cognitive status, Medicaid enrollment, discharge diagnosis, and balance while walking were most predictive of reduced odds of community discharge.

### Model Evaluation

**Fig. 1.**
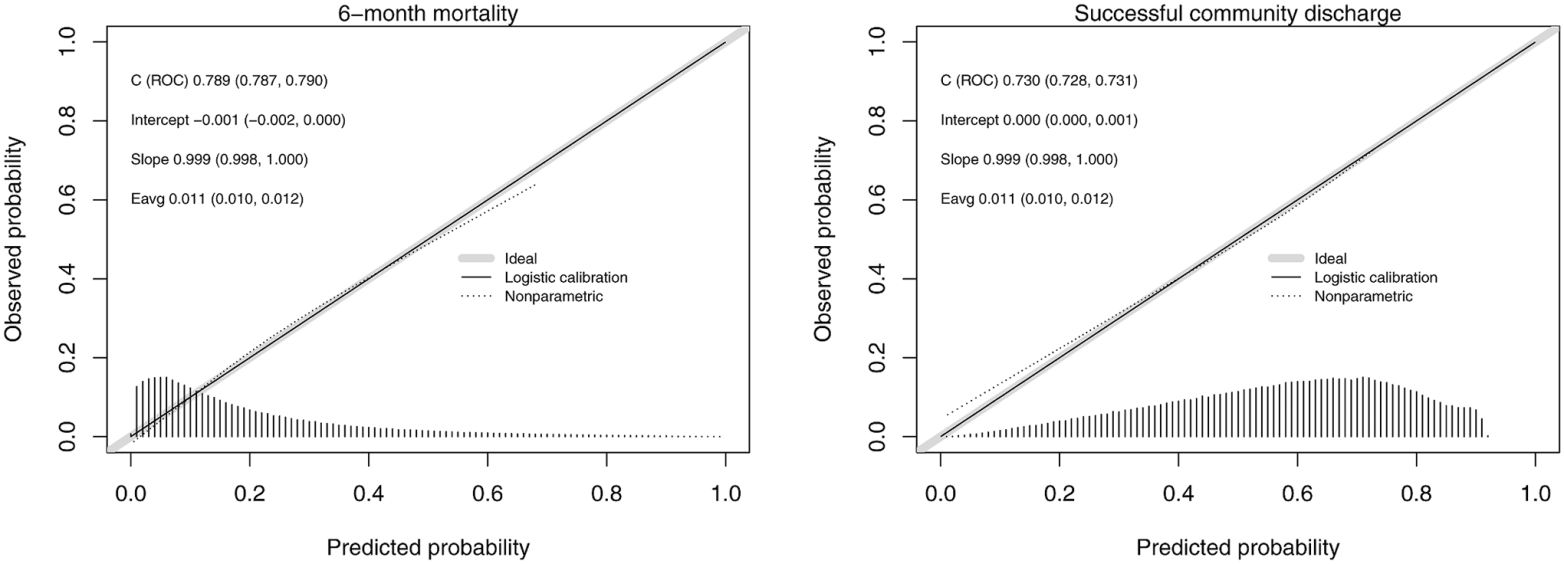
Model performance and calibration plots for the updated MDS model in predicting 6-month mortality and successful community discharge among older adults admitted to an SNF for short-term post-acute care. Calibration plots indicate the agreement between the predicted risk of the outcome using the prediction model and the observed risk of the outcome. Perfect predictions would be at the 45-degree line. Each plot also contains a density plot placed on the x-axis displaying the distribution of predicted risk in the cohort.

When predicting 6-month mortality, the optimism-corrected c-statistics after bootstrap internal validation were 0.747 (95% CI, 0.745–0.749) for the Medicare base model and 0.789 (95% CI, 0.787–0.790) for the updated MDS model (Table 3, Supplementary Table 10). Both the base model and updated MDS models were well calibrated for both outcomes with good agreement between observed and predicted risk (Figure 1, Table 3, Supplementary Figure 4). When predicting successful community discharge, the optimism-corrected c-statistics were 0.685 (95% CI, 0.683–0.687) for the Medicare base model and 0.730 (95% CI, 0.728–0.731) for the updated MDS model (Table 3, Supplementary Table 10). The models were similarly well calibrated (Figure 1, Table 3, Supplementary Figure 4).

For 6-month mortality, decision curve analysis showed that the updated MDS model had greater net benefit across clinically relevant risk thresholds between ~15% and 60% compared with using the base Medicare model, a simplified model with age and sex, and the intervene for all and intervene for none strategies (Supplementary Figure 5). For a scenario in which an SNF is willing to flag patients for comprehensive care conferences or palliative care referrals at a threshold of 30% to 50% for dying within 6 months, use of the MDS model would result in a net benefit of 0.02 to 0.04. This is higher than the strategy of intervening for no patients (net benefit of 0) or all patients (a harmful strategy in which the net benefit is −0.233 to −0.781), indicating increased clinical utility. A net benefit of 0.02 to 0.04 indicates that using the model helps to correctly identify an additional 2 to 4 patients of 100 who will die at 6 months over and above those falsely identified as being at high risk. For successful community discharge, the updated MDS model showed greater net benefit at risk thresholds between ~35% and 80% (Supplementary Figure 6).

Compared with the base Medicare model, the updated MDS model showed increased variance in predicted risks for both outcomes, as demonstrated by a widening in the histogram of risk predictions (Supplementary Figures 7 and 8). In addition, the MDS model provided updated risk estimates that could vary significantly for certain patients compared with the base Medicare model (Supplementary Figures 9 and 10). The fraction of total diagnostic information provided by MDS elements when compared with the base Medicare model was ~0.3 and ~0.37 for 6-month mortality and successful community discharge, respectively (Supplementary Table 11). This indicates that ~30% of the total predictive information across all predictors was added by the MDS data elements.

In a secondary analysis, model discrimination and calibration were largely similar across subgroups by age, sex, race/ethnicity, and hospital discharge diagnosis (Supplementary Tables 12 and 13). Although there was a range of discrimination across subgroups (c-statistic of 0.866 in the “Musculoskeletal” hospital discharge diagnosis subgroup vs 0.745 in the “Other” hospital discharge diagnosis subgroup for 6-month mortality), the discrimination of most subgroups clustered around the overall c-statistics. We plan to host the updated MDS model on the ePrognosis website.^7^ The Supplementary Methods show the full equations for the model and examples of how to compute risk estimates. Table 4 shows the probability of 6-month mortality and successful community discharge for 10 randomly selected individuals.

## Discussion

We developed and internally validated an updated prognostic model for 6-month mortality and successful community discharge for community-dwelling older adults admitted for post-acute care at SNFs that incorporates MDS data elements. Inclusion of MDS data elements improved discrimination, showed additional net benefit, and provided additional diagnostic information compared with using only Medicare data. SNF clinicians can use the updated MDS model to produce more accurate prognostic information 1 week into a post-hospitalization SNF stay.

Other prediction models have been published among short-stay and long-stay residents using MDS data.^33^ Indices among long-stay residents include the Flacker-Kiely models, MDS Mortality Risk Index-Revised (MMRI-R), its updated version using MDS 3.0 variables (MMRI-R-v3), the MDS Changes in Health, End-stage disease and Symptoms and Signs (CHESS), its updated version using MDS 3.0 variables (MDS-CHESS 3.0), and the MDS 3.0 Mortality Risk Score (MRS3).^14–16,18,34,35^ These models typically include variables such as age, functional status, clinical signs and symptoms (eg, shortness of breath, dehydration, pressure ulcers), and cognitive impairment. The c-statistics for mortality generally were around 0.71.^33^ Calibration was not always reported. One model, which assessed successful community discharge, showed a c-statistic of 0.614.^15^ The primary advantage of our model is that we used updated data, included a more specific post-acute care cohort (ie, the cohorts from these prior models either included only long-stay residents or included a mixture of short-stay and long-stay residents), and provided a web-based calculator to facilitate the model’s use in clinical practice.

The most directly comparable model for short-stay SNF patients was developed by Burke et al,^19^ involving 2043 individuals from the Medicare Current Beneficiary Survey (2003–2011). The model included MDS elements (Barthel index, Charlson-Deyo score, inpatient length of stay, presence of catheter, heart failure) to predict a composite of hospital readmission, SNF stay >100 days, and death during SNF stay. The optimism-corrected c-statistic was 0.75, and the model showed good calibration. Advantages of our model include the use of more recent data with a larger sample size and choosing not to predict a composite outcome, which allows for coefficients to vary based on the 2 outcomes.

The updated MDS model can be implemented into real-world workflows in a variety of ways, such as during multidisciplinary care conferences that occur a few days into the SNF admission when patient health trajectories are more established. Older adults with serious illness and limited life expectancy are frequently discharged from the hospital to SNFs with overly optimistic estimates about their health trajectory and potential for functional recovery.^36–38^ Given the challenges in engaging in difficult goals-of-care discussions, this can lead to repeated cycles of hospitalizations and SNF admissions that may not be aligned with patient values and preferences.^5,6,39^ Prognostic estimates of 6-month mortality can help frame goals of care and advance care planning discussions and flag individuals for palliative care and hospice referrals to ensure that care provided is concordant with patient goals.^39^ Risk estimates can also prompt SNF clinicians to re-address chronic disease management (eg, overly aggressive treatment of diabetes and hypertension in which the harms of strict glycemic or blood pressure control outweigh the benefits).^40^ Estimates of successful community discharge can help set expectations around recovery and prompt conversations with case managers about additional resources to support a home discharge (eg, home modifications, hiring paid caregivers) or prepare for a long-term care admission (eg, completing Medicaid application, investigating local facilities).

For SNF clinicians who wish to obtain risk estimates immediately on SNF admission, use of the previously published Medicare-only model is preferable, as it was specifically developed to provide predictions on day 1 of SNF admission.^2^ The updated MDS model most directly applies to patients who remain at an SNF for at least 7 days (ie, have not been re-admitted to the hospital or discharged before day 7). If clinicians do not have access to the exact scores for functional status based on the MDS-ADL, they can use clinical judgment in entering these values (eg, an MDS-ADL score of 21 indicates extensive assistance with all activities). In addition, clinicians should note that cognitive status on admission does not necessarily reflect an individual’s cognitive status before the hospitalization (eg, due to the presence of delirium).

### Strengths and Limitations

A strength of our study is the use of a large 20% national Medicare sample to develop prognostic models for 2 clinically relevant outcomes, whereby predictions can be obtained through an online calculator. Our study has a few limitations. First, we only included Medicare Fee-for-Service beneficiaries within the United States. Predictions from this model may not apply to individuals enrolled in Medicare Advantage, who may experience differences in insurance coverage determinations due to prior authorization denials, narrower SNF networks, shorter SNF lengths of stay, and potentially higher likelihood of successful community discharge.^41–45^ Similarly, predictions may not apply to those outside the United States because of differences in post-acute and long-term care systems.^46^ Future studies are needed to perform an external validation if used in different populations. Second, community discharges are ultimately influenced by many factors not included in this model (eg, presence of family or other caregivers, neighborhood environment, and attitudes around long-term care). Because potentially important patient-level factors could not be incorporated into our models, clinician judgment remains critical in interpreting quantitative predictions from our model.

## Conclusions and Implications

We developed and internally validated an updated prognostic model for 6-month mortality and community discharge for short-stay SNF patients that incorporated MDS data elements to improve model performance. Through an online calculator, SNF clinicians can use risk estimates to frame discussions during a post-acute SNF stay around advance care planning, palliative care referrals, management of chronic conditions, and discharge planning.

## Supplementary Material

1

Supplementary Data

Supplementary data related to this article can be found online at https://doi.org/10.1016/j.jamda.2025.106103.

## Figures and Tables

**Table 1:** Baseline Characteristics of Individuals Admitted for Short-Term Rehab at an SNF Between 2017 and 2019 Who Remain in a Facility on Day 7*

Characteristic	Overall (n = 426,680)	6-Month Mortality Outcome		Successful Community Discharge Outcome	
		Alive (n = 341,966)	Died (n = 84,714)	Successful Community Discharge (n = 245,591)	Did Not Experience a Successful Community Discharge (n = 181,189)
Age, y, mean (SD)	81.3 (8.26)	80.8 (8.17)	83.2 (8.35)	80.8 (8.11)	82.0 (8.42)
Sex					
Male	159,291 (37.3)	120,307 (35.2)	38,984 (46.0)	86,056 (35.1)	73,235 (40.4)
Female	267,389 (62.7)	221,659 (64.8)	45,730 (54.0)	159,435 (64.9)	107,954 (59.6)
Race and ethnicity					
Non-Hispanic Black	33,502 (7.9)	26,625 (7.8)	6877 (8.1)	17,444 (7.1)	16,058 (8.9)
Hispanic	16,173 (3.8)	13,208 (3.9)	2965 (3.5)	8923 (3.6)	7250 (4.0)
Non-Hispanic White	362,591 (85.0)	290,243 (84.9)	72,348 (85.4)	210,809 (85.9)	151,782 (83.8)
Other^†^	14,414 (3.4)	11,890 (3.5)	2524 (3.0)	8315 (3.4)	6099 (3.4)
Medicaid enrolled	76,183 (17.9)	61,224 (17.9)	14,959 (17.7)	34,518 (14.1)	41,665 (23.0)
Comorbidities					
Weight loss	74,712 (17.5)	50,227 (14.7)	24,485 (28.9)	33,532 (13.7)	41,180 (22.7)
Heart failure	154,437 (36.2)	112,975 (33.0)	41,462 (48.9)	78,231 (31.9)	76,206 (42.1)
Diabetes (complicated)	130,844 (30.7)	102,828 (30.1)	28,016 (33.1)	70,706 (28.8)	60,138 (33.2)
Renal failure (severe)	39,361 (9.2)	26,766 (7.8)	12,595 (14.9)	17,797 (7.2)	21,564 (11.9)
Liver failure (severe)	5644 (1.3)	3391 (1.0)	2253 (2.7)	2418 (1.0)	3226 (1.8)
Dementia	118,818 (27.8)	89,184 (26.1)	29,634 (35.0)	54,458 (22.2)	64,360 (35.5)
Other neurologic disease	92,971 (21.8)	69,221 (20.2)	23,750 (28.0)	43,984 (17.9)	48,987 (27.0)
Lung disease	164,566 (38.6)	12,5741 (36.8)	38,825 (45.8)	89,626 (36.5)	74,940 (41.4)
Paralysis	41,327 (9.7)	32,342 (9.5)	8985 (10.6)	20,140 (8.2)	21,187 (11.7)
Metastatic cancer	18,928 (4.4)	8315 (2.4)	10,613 (12.5)	7296 (3.0)	11,632 (6.4)
Solid cancer	52,132 (12.2)	39,361 (11.5)	12,771 (15.1)	29,216 (11.9)	22,916 (12.6)
Leukemia/lymphoma	13,258 (3.1)	8881 (2.6)	4377 (5.2)	6610 (2.7)	6648 (3.7)
Hospital discharge diagnosis					
Injury (eg, fracture)	100,350 (23.5)	86,521 (25.3)	13,829 (16.3)	62,765 (25.6)	37,585 (20.7)
Circulatory (eg, heart failure, stroke)	76,340 (17.9)	58,023 (17.0)	18,317 (21.6)	40,339 (16.4)	36,001 (19.9)
Musculoskeletal (eg, knee replacement)	52,411 (12.3)	48,925 (14.3)	3486 (4.1)	40,748 (16.6)	11,663 (6.4)
Neoplastic	12,693 (3.0)	6765 (2.0)	5928 (7.0)	5531 (2.3)	7162 (4.0)
Other	184,886 (43.3)	141,732 (41.4)	43,154 (50.9)	96,108 (39.1)	88,778 (49.0)
Hospital length of stay, mean (SD)	8.20 (5.68)	7.93 (5.50)	9.31 (6.21)	7.48 (5.00)	9.18 (6.35)
Hospitalizations in the past year					
0	264,981 (62.1)	219,772 (64.3)	45,209 (53.4)	162,508 (66.2)	102,473 (56.6)
1	93,887 (22.0)	73,320 (21.4)	20,567 (24.3)	51,847 (21.1)	42,040 (23.2)
2	36,661 (8.6)	27,194 (8.0)	9467 (11.2)	18,114 (7.4)	18,547 (10.2)
3	16,038 (3.8)	11,379 (3.3)	4659 (5.5)	7294 (3.0)	8744 (4.8)
≥4	15,113 (3.5)	10,301 (3.0)	4812 (5.7)	5728 (2.3)	9385 (5.2)
ADL score, mean (SD) (range 0–28)^‡^	17.0 (3.99)	16.7 (3.99)	18.4 (3.69)	16.3 (3.96)	18.0 (3.83)
Cognitive status^§^					
No impairment	264,125 (61.9)	225,081 (65.8)	39,044 (46.1)	173,149 (70.5)	90,976 (50.2)
Mild impairment	89,581 (21.0)	68,461 (20.0)	21,120 (24.9)	45,546 (18.6)	44,035 (24.3)
Moderate impairment	61,315 (14.4)	41,857 (12.2)	19,458 (23.0)	23,404 (9.5)	37,911 (20.9)
Severe impairment	8452 (2.0)	4369 (1.3)	4083 (4.8)	1913 (0.8)	6539 (3.6)
Missing	3207 (0.8)	2198 (0.6)	1009 (1.2)	1479 (0.6)	1728 (1.0)
Oxygen use					
Yes	94,386 (22.1)	65,284 (19.1)	29,102 (34.4)	45,906 (18.7)	48,480 (26.8)
No	332,160 (77.8)	276,569 (80.9)	55,591 (65.6)	199,506 (81.3)	132,654 (73.2)
Missing	134 (0.0)	113 (0.0)	21 (0.0)	79 (0.0)	55 (0.0)
Urinary incontinence					
Always continent	147,524 (34.6)	129,623 (37.9)	17,901 (21.1)	104,285 (42.5)	43,239 (23.9)
Occasionally incontinent	122,524 (28.7)	100,891 (29.5)	21,633 (25.5)	74,477 (30.3)	48,047 (26.5)
Frequently incontinent	95,561 (22.4)	71,380 (20.9)	24,181 (28.5)	44,613 (18.2)	50,948 (28.1)
Always incontinent	29,929 (7.0)	18,892 (5.5)	11,037 (13.0)	9320 (3.8)	20,609 (11.4)
Not rated (eg, urinary catheter)	31,034 (7.3)	21,104 (6.2)	9930 (11.7)	12,754 (5.2)	18,280 (10.1)
Missing	108 (0.0)	76 (0.0)	32 (0.0)	42 (0.0)	66 (0.0)
Bowel incontinence					
Always continent	230,044 (53.9)	198,438 (58.0)	31,606 (37.3)	154,668 (63.0)	75,376 (41.6)
Occasionally incontinent	57,019 (13.4)	45,523 (13.3)	11,496 (13.6)	32,136 (13.1)	24,883 (13.7)
Frequently incontinent	89,989 (21.1)	66,244 (19.4)	23,745 (28.0)	41,805 (17.0)	48,184 (26.6)
Always incontinent	40,478 (9.5)	24,878 (7.3)	15,600 (18.4)	12,215 (5.0)	28,263 (15.6)
Not rated (eg, ostomy)	8962 (2.1)	6746 (2.0)	2216 (2.6)	4570 (1.9)	4392 (2.4)
Missing	188 (0.0)	137 (0.0)	51 (0.1)	97 (0.0)	91 (0.1)
Balance while walking					
Steady at all times	13,083 (3.1)	11,710 (3.4)	1373 (1.6)	9125 (3.7)	3958 (2.2)
Not steady but stabilizes without assistance	79,355 (18.6)	69,371 (20.3)	9984 (11.8)	55,046 (22.4)	24,309 (13.4)
Not steady but stabilizes with assistance	231,248 (54.2)	187,790 (54.9)	43,458 (51.3)	139,007 (56.6)	92,241 (50.9)
Did not walk	101,633 (23.8)	72,063 (21.1)	29,570 (34.9)	41,620 (17.0)	60,013 (33.1)
Missing	1361 (0.3)	1032 (0.3)	329 (0.4)	693 (0.3)	668 (0.4)

Values are n (%) unless otherwise indicated.

* A full description of baseline characteristics for the cohort can be found in Supplementary Table 5. Percentages represent column percentages for each characteristic.

† The other category includes American Indian, Alaska Native, Asian, Native Hawaiian, Pacific Islander, and individuals with multiple race categories. Race and ethnicity was not included as a candidate predictor for model development.

‡ The MDS-ADL scale assesses 7 ADL items, including bed mobility, transfers, locomotion on unit, dressing, eating, toilet use, and personal hygiene. For each ADL, the individual is given a score from 0 (independent) to 4 (total dependence). Scores on the MDS-ADL scale range from 0 to 28, with higher scores indicating increased functional impairment. See the Supplementary Methods for details. For this variable, a total of 345 (0.1%) individuals in our cohort had missing values.

§ Cognitive status is based on the Cognitive Function Scale (CFS), which uses the Brief Interview for Mental Status (BIMS) screener (scored from 0 to 15) or Cognitive Performance Scale (CPS) (scored from 0 to 6). Staff will conduct the CPS if the individual is deemed inappropriate or unable to complete the BIMS screener. The CFS is scored as follows: cognitively intact (BIMS score 13–15), mildly impaired (BIMS score of 8–12 or CPS score of 0–2), moderately impaired (BIMS score of 0–7 or CPS score of 3–4), and severely impaired (CPS score of 5–6).

**Table 2:** Multivariable-Adjusted Odds Ratios for Predictors Included in the Final Updated MDS Model for the Outcomes of 6-Month Mortality and Successful Community Discharge

Predictor	6-Month Mortality	Successful Community Discharge
	OR (95% CI)	OR (95% CI)
Age (OR from age of 75 to 88)*	1.60 (1.56–1.64)	0.84 (0.82–0.85)
Sex (male vs female)	1.40 (1.38–1.43)	0.90 (0.89–0.91)
Medicaid eligible (yes vs no)	0.80 (0.79–0.82)	0.65 (0.64–0.66)
Comorbidities		
Weight loss	1.62 (1.58–1.65)	0.81 (0.79–0.82)
Heart failure	1.45 (1.42–1.47)	0.84 (0.83–0.86)
Diabetes (complicated)	1.03 (1.01–1.05)	0.95 (0.94–0.97)
Renal failure (severe)	1.69 (1.64–1.73)	0.76 (0.75–0.78)
Liver failure (severe)	2.98 (2.80–3.16)	0.63 (0.59–0.66)
Dementia	0.97 (0.95–0.99)	0.83 (0.82–0.85)
Other neurologic disease	0.97 (0.95–0.99)	1.00 (0.98–1.01)
Lung disease	1.22 (1.20–1.24)	0.93 (0.91–0.94)
Paralysis	0.78 (0.76–0.81)	1.12 (1.09–1.14)
Metastatic cancer	6.00 (5.78–6.22)	0.49 (0.48–0.51)
Solid cancer	1.39 (1.35–1.42)	0.93 (0.91–0.95)
Leukemia/lymphoma	1.67 (1.60–1.74)	0.80 (0.77–0.83)
Principal discharge diagnosis		
Injury (eg, fractures)	0.62 (0.61–0.64)	1.33 (1.31–1.36)
Circulatory (eg, heart failure, stroke)	1.14 (1.11–1.17)	0.95 (0.93–0.97)
Musculoskeletal	0.59 (0.57–0.61)	1.45 (1.42–1.49)
Neoplasms	2.15 (2.05–2.25)	0.72 (0.69–0.75)
Other	Ref	Ref
Admission type (urgent/emergent vs elective)	1.71 (1.66–1.76)	0.73 (0.72–0.75)
Hospital length of stay (OR from length of stay of 4 to 9 days)*	1.15 (1.12–1.17)	0.83 (0.82–0.84)
Hospitalizations in the past year		
0	Ref	Ref
1	1.12 (1.09–1.14)	0.90 (0.89–0.92)
2	1.23 (1.19–1.27)	0.77 (0.75–0.79)
3	1.35 (1.30–1.41)	0.70 (0.68–0.73)
4+	1.38 (1.33–1.44)	0.56 (0.54–0.58)
MDS-ADL score (OR from score of 15 to 19)*^,†^	1.28 (1.25–1.31)	0.82 (0.80–0.83)
Cognitive status^‡^		
No impairment	Ref	Ref
Mild impairment	1.39 (1.36–1.43)	0.74 (0.72–0.75)
Moderate impairment	1.97 (1.91–2.02)	0.56 (0.55–0.57)
Severe impairment	2.92 (2.77–3.08)	0.43 (0.41–0.45)
Oxygen use (yes vs no)	1.73 (1.70–1.76)	0.79 (0.77–0.80)
Urinary incontinence		
Always continent	Ref	Ref
Occasionally incontinent	1.11 (1.08–1.14)	0.85 (0.84–0.87)
Frequently incontinent	1.18 (1.15–1.22)	0.74 (0.72–0.76)
Always incontinent	1.20 (1.15–1.26)	0.72 (0.69–0.75)
Not rated (eg, urinary catheter)	1.40 (1.35–1.45)	0.62 (0.60–0.64)
Bowel incontinence		
Always continent	Ref	Ref
Occasionally incontinent	1.15 (1.12–1.18)	0.89 (0.88–0.91)
Frequently incontinent	1.29 (1.25–1.32)	0.83 (0.81–0.84)
Always incontinent	1.56 (1.50–1.62)	0.69 (0.67–0.71)
Not rated (eg, ostomy)	1.12 (1.06–1.19)	0.88 (0.84–0.93)
Balance while walking		
Steady at all times	Ref	Ref
Not steady but stabilizes without assistance	1.06 (0.99–1.13)	1.07 (1.03–1.12)
Not steady but stabilizes with assistance	1.20 (1.12–1.28)	0.97 (0.93–1.01)
Did not walk	1.52 (1.42–1.63)	0.66 (0.63–0.69)

OR, Odds ratio; Ref, reference group.

* Age was modeled continuously as a restricted cubic spline with 4 knots placed at ages 68, 78, 85, and 94 years. Hospital length of stay was modeled continuously as a restricted cubic spline with 4 knots placed at 4, 5, 8, and 21 days. ADL score was modeled continuously as a restricted cubic spline with 4 knots placed at 9, 17, 19, and 22. See the Supplementary Methods for the full model formula and Supplementary Tables 2–4 for detailed descriptions of the individual variables.

† The MDS-ADL score ranges from 0 to 28 with higher scores indicating increased dependence in functioning. Individuals are scored from 0 (independent) to 4 (total dependence) on 7 ADLs, including bed mobility, transfer, locomotion on unit, dressing, eating, toileting, and personal hygiene.

‡ Cognitive status is based on the Cognitive Function Scale (CFS), which uses the Brief Interview for Mental Status (BIMS) screener (scored from 0 to 15) or Cognitive Performance Scale (CPS) (scored from 0 to 6). Staff will conduct the CPS if the individual is deemed inappropriate or unable to complete the BIMS screener. The CFS is scored as follows: cognitively intact (BIMS score 13–15), mildly impaired (BIMS score of 8–12 or CPS score of 0–2), moderately impaired (BIMS score of 0–7 or CPS score of 3–4), and severely impaired (CPS score of 5–6).

**Table 3:** Optimism-Corrected Model Performance for Predicting 6-Month Mortality and Successful Community Discharge Following Bootstrap Internal Validation

Performance Measure	Day 7 Base Model Without MDS Predictors	Day 7 MDS Model
6-Month Mortality		
c-statistic	0.747 (0.745 to 0.749)	0.789 (0.787 to 0.790)
Calibration in the large (intercept)	−0.001 (−0.003 to 0.001)	−0.001 (−0.002 to 0.000)
Calibration slope	0.999 (0.998 to 1.001)	0.999 (0.998 to 1.000)
ICI (Eavg)	0.012 (0.011 to 0.012)	0.011 (0.010 to 0.012)
Brier score	0.139 (0.138 to 0.140)	0.130 (0.130 to 0.131)
Successful community discharge		
c-statistic	0.685 (0.683 to 0.687)	0.730 (0.728 to 0.731)
Calibration in the large (intercept)	0.000 (−0.001 to 0.001)	0.000 (0.000 to 0.001)
Calibration slope	0.999 (0.998 to 1.000)	0.999 (0.998 to 1.000)
ICI (Eavg)	0.013 (0.012 to 0.014)	0.011 (0.010 to 0.012)
Brier score	0.219 (0.219 to 0.220)	0.206 (0.206 to 0.207)

**Table 4:** Baseline Characteristics and Predicted Risk of 6-Month Mortality and Successful Community Discharge for 10 Individuals Based on Quintiles of Predicted Risk*

Individual	1	2	3	4	5	6	7	8	9	10
Characteristic										
Age, y	85	72	83	81	85	68	85	87	69	69
Sex	Female	Male	Female	Female	Male	Female	Female	Female	Female	Male
Medicaid enrolled	No	No	No	No	No	No	No	No	No	No
Comorbidities	None	Lung disease, other neurologic disease	Heart failure	Dementia	Weight loss, diabetes (complicated), leukemia/lymphoma, solid cancer	Weight loss, solid cancer	Heart failure, paralysis	Heart failure, renal failure (severe)	Weight loss, lung disease	Diabetes (complicated)
Discharge diagnosis	MSK	Injury	Other	Injury	Neoplasm	Other	Circulatory	Injury	Other	Circulatory
Hospital length of stay, d	4	8	9	5	19	15	5	5	10	6
Admission type	Elective	Urgent	Urgent	Urgent	Urgent	Elective	Urgent	Urgent	Urgent	Elective
Hospitalizations in the past year	0	0	0	0	0	1	0	2	1	0
MDS-ADL score, range 0–28	19	19	17	23	15	20	21	18	10	11
Cognitive status	Intact	Intact	Intact	Severe impairment	Intact	Severe impairment	Mild impairment	Intact	Intact	Intact
Urinary incontinence	Occasionally incontinent	Frequently incontinent	Always continent	Always incontinent	Frequently incontinent	Always incontinent	Frequently incontinent	Frequently incontinent	Always continent	Always continent
Bowel incontinence	Always continent	Frequently incontinent	Always continent	Always incontinent	Occasionally incontinent	Always incontinent	Frequently incontinent	Frequently incontinent	Always continent	Always continent
Oxygen use	No	No	Yes	No	No	No	No	No	Yes	Yes
Balance with walking	Not steady, stabilizes with assistance	Not steady, stabilizes with assistance	Not steady but stabilizes without assistance	Not steady, stabilizes with assistance	Not steady, stabilizes with assistance	Not steady, stabilizes with assistance	Did not walk	Not steady, stabilizes with assistance	Not steady but stabilizes without assistance	Not steady, stabilizes with assistance
6-month mortality, %	2.7	8.0	13.0	22.9	50.7	31.5	23.7	18.1	10.9	5.4
Successful community discharge, %	85.8	69.8	71.9	39.6	41.5	34.2	45.2	58.5	71.0	80.5

MSK, Musculoskeletal.

* Individuals 1–5 were randomly selected within each fifth of the predicted risk for 6-month mortality. Individuals 6–10 were randomly selected within each fifth of the predicted risk for successful community discharge. The probability of 6-month mortality and successful community discharge, as estimated by the prognostic model, is provided in the 2 bottom rows.
