# Supplementary material for: An Updated Mortality and Community Discharge Prognostic Model for Older Adults Admitted to Skilled Nursing Facilities for Post-Acute Care
Source: J Am Med Dir Assoc. Author manuscript; Available in PMC 2026 Apr 12. (PMC13070420; doi:10.1016/j.jamda.2025.106103)
Supplement: 1 [file NIHMS2163403-supplement-1.docx]

**Supplementary Materials**

**Supplementary Methods**

**Supplementary Figure S1**: Flow diagram for cohort construction

**Supplementary Figure S2**: Plot of the proportion of overall X2 for each predictor in the Updated MDS model when predicting 6-month mortality for older adults admitted to a skilled nursing facility

**Supplementary Figure S3:** Plot of the proportion of overall X2 for each predictor in the Updated MDS model when predicting successful community discharge for older adults admitted to a skilled nursing facility

**Supplementary Figure S4**: Calibration plot of the base model in predicting 6-month mortality and successful community discharge

**Supplementary Figure S5:** Decision curve analysis for the outcome of 6-month mortality comparing the net benefit of the base model, updated Minimum Data Set model, a simplified model including only age and sex, and the intervene for all and intervene for none strategies across a range of thresholds

**Supplementary Figure S6:** Decision curve analysis for the outcome of successful community discharge comparing the net benefit of the base model, updated Minimum Data Set model, a simplified model including only age and sex, and the intervene for all and intervene for none strategies across a range of thresholds

**Supplementary Figure S7:** Back-to-back histogram of predicted risks for the base Medicare model and the updated MDS model when predicting 6-month mortality

**Supplementary Figure S8:** Back-to-back histogram of predicted risks for the base Medicare model and the updated MDS model when predicting successful community discharge

**Supplementary Figure S9:** Plot of the estimated probability of 6-month mortality for individuals using predictions from the base Medicare model and the updated MDS model

**Supplementary Figure S10:** Plot of the estimated probability of successful community discharge for individuals using predictions from the base Medicare model and the updated MDS model

**Supplementary Table S1:** TRIPOD+AI Checklist

**Supplementary Table S2:** Variables included in the base model, including specification

**Supplementary Table S3:** Categorization of principal hospital discharge diagnoses according to the Clinical Classifications Software Refined (CCSR) for ICD-10 diagnoses

**Supplementary Table S4:** Candidate predictor variables considered for inclusion in the updated Minimum Data Set model, including specification

**Supplementary Table S5**: Baseline characteristics of individuals admitted to a skilled nursing facility for post-acute care from 2017-2019 who remained at a skilled nursing facility on day 7 and had a completed Minimum Data Set admission assessment

**Supplementary Table S6:** Univariable association between predictors and 6-month mortality for older adults admitted to a skilled nursing facility

**Supplementary Table S7:** Univariable association between predictors and successful community discharge for older adults admitted to a skilled nursing facility

**Supplementary Table S8:** Full model coefficients for the final model for predicting 6-month mortality for older adults admitted to a skilled nursing facility following a hip fracture

**Supplementary Table S9:** Full model coefficients for the final model for predicting successful community discharge for older adults admitted to a skilled nursing facility following a hip fracture

**Supplementary Table S10.** Apparent and optimism-corrected performance following bootstrap internal validation of the base model and updated Minimum Data Set model for predicting 6-month mortality and successful community discharge

**Supplementary Table S11:** Additional performance measures to assess the value of adding Minimum Data Set data elements to the base model

**Supplementary Table S12:** Optimism-corrected performance of the model for the outcome of 6-month mortality across subgroups by age, sex, race and ethnicity, and clinical diagnosis

**Supplementary Table S13:** Optimism-corrected performance of the model for the outcome of successful community discharge across subgroups by age, sex, race and ethnicity, and clinical diagnosis

**Supplementary Methods**

Cohort construction

Using data from a 20% national sample of all Medicare beneficiaries, we first created a cohort of community-dwelling older adults aged ≥66 discharged to a skilled nursing facility (SNF) following a hospitalization between 2017-2019. We excluded individuals <66 years of age (to establish a 1-year look-back period for information on comorbidities and prior healthcare use), those who did not have continuous Medicare Fee-for-Service (FFS) coverage (as individuals enrolled in Medicare Advantage have different insurance coverage determinations in post-acute care), individuals residing in a nursing home for long-term care in the 3 months prior to SNF admission (given our focus on community-dwelling older adults), and individuals who were not in the community directly before the index hospitalization (e.g., admitted directly to the hospital from a SNF, acute rehab, or long-term acute care hospital). We subsequently refined our cohort to include individuals who remained at a SNF on day 7 and had a comprehensive MDS assessment on or before day 7 of admission. These choices reflected our goal to develop an index for use on day 7 of SNF admission when clinical trajectories are more well-established and incorporate MDS data elements which are often completed a few days after SNF admission and may require a 7-day lookback period for full assessment.

There are a few different types of MDS assessments, including comprehensive item sets (NC), quarterly item sets (NQ), Prospective Payment System item sets (NP), discharge item sets (ND), and tracking item sets (NT). For short-stay residents, the entry tracking record (NT) is the first item set completed for all residents and contains information about whether this is the individual’s first entry into a facility (admission) or if it represents a readmission (reentry). We used the MDS NT item set to refine our cohort to only include individuals who were newly admitted to a SNF. We additionally only included individuals where the MDS indicated that the admission was from a hospital to exclude individuals being admitted from psychiatric hospitalizations, inpatient rehab, long-term acute care hospitals, or hospice.

For short-stay residents, the first comprehensive admission MDS assessment which contains information on functional status, cognition, and other items usually involves the NC or NP item set. For short-stay residents receiving care under Medicare Part A, the Prospective Payment System assessments using the NP item set are required for reimbursement. The first assessment is known as the 5-day assessment and must be completed within 14 days after an assessment reference date set by the SNF. However, nursing homes are also required by the Omnibus Budget Reconciliation Act of 1987 (OBRA) to complete other MDS assessments (such as the comprehensive NC assessment). For individuals receiving short-term care at SNFs under Medicare Part A, the 5-day admission PPS assessment may be combined with the OBRA admission assessment. Therefore, we included individuals with either an NP or NC assessment. We excluded individuals whose first comprehensive assessment was noted as a discharge assessment as these were individuals with typically very short SNF stays who would likely not as benefit as much from prognostic conversations. After applying our inclusion and exclusion criteria, we were able to construct a cohort of individuals with an initial MDS tracking record indicating admission into a SNF with a second assessment of NC or NP type indicating an OBRA and/or PPS admission assessment within 7 days of SNF admission which was not a discharge assessment.

Sample size calculation

We used the pmsampsize package in Stata to determine whether our sample size was sufficient to ensure key parameters were estimated precisely and to minimize overfitting. We calculated the minimum sample size required for each outcome. For 6-month mortality, we used a binary logistic regression model with 76 candidate predictor parameters, 0.9 level of shrinkage, 0.20 as outcome proportion, and 0.75 as the c-statistic reported previously. The minimum sample size required would be ~5,000 individuals which is much smaller than our cohort size. For successful community discharge, we used a binary logistic regression model with 76 candidate predictor parameters, 0.9 level of shrinkage, 0.50 as the outcome proportion, and 0.68 as the c-statistic reported previously. The minimum sample size required would be ~6,500 individuals which is much smaller than our cohort size.

Missing data

We did not have any missing data for the Medicare-derived variables (age, sex, Medicaid enrollment, comorbidities, hospital discharge diagnosis, hospital length of stay, admission type, number of hospitalizations in the past year). However, we did have some missingness with the MDS elements which were all less than 5%. Variables with the largest percentage of missingness were falls (5%), marital status (4%), delirium (3%), body mass index (2%), pain (1%), depression (1%), and cognitive score (1%). All other MDS variables had <1% missingness. We found that roughly 85% of individuals in our cohort had no missing information on any of the MDS candidate variables.

To address the issue of missing data, we created 20 imputed datasets using predictive mean matching multiple imputation. We then created a combined dataset by choosing the modal value for categorical variables and mean value for numeric variables (MDS-ADL score and body mass index) among the 20 imputed datasets. We performed analyses for variable selection using LASSO in both the imputed datasets and the modal dataset and found similar results. To determine the final coefficients of the model and measures of model performance (e.g., discrimination and calibration), we used the imputed datasets and pooled the results. For the analyses involving the decision curves and fraction of new information provided, we used the modal dataset.

Variable selection with the MDS data elements

We considered several predictors from the admission MDS assessment to include in the Updated MDS model (Supplementary Table S4). These included items such as marital status, cognitive function scale, functional status (based on the MDS-Activities of Daily Living long-form scale), balance while walking, delirium (based on the Confusion Assessment Method), mood, psychosis, behavioral issues (based on the Agitated and Reactive Behavior Scale), urinary and bowel incontinence, shortness of breath, falls history, dysphagia, body mass index, pressure ulcers, oxygen use, intravenous medications, dialysis, chemotherapy, radiation therapy, pain, and receipt of anticoagulants, antipsychotics, hypnotics, or diuretics.

To facilitate the adoption of our prognostic model in clinical practice by busy clinicians, we performed variable selection on the MDS data elements to create a more parsimonious model. We pre-specified the predictors from the base Medicare model that would stay in the MDS model (age, sex, Medicaid eligibility, hospital length of stay, hospital discharge diagnosis, admission type, hospitalizations in the past year, comorbidities). We then performed variable selection on the MDS predictors via Least Absolute Shrinkage and Selection Operator (LASSO) using the glmnet package in R on each outcome separately (6-month mortality and successful community discharge). We used LASSO to shrink the coefficients of the MDS data elements. Due to the small amount of missing data, we ran LASSO on the 20 imputed datasets and the combined modal dataset. Given the size of our cohort, the coefficients for several of these variables were not shrunk to 0 and remained in the model. To come up with a list of roughly 5-6 MDS data elements that had the most prognostic power, we identified the sequence in which predictors were included in the LASSO procedure by checking variables selected at each lambda for each outcome separately. We found that the order of variables selected were stable across the 20 imputed datasets and the combined modal dataset.

For predicting 6-month mortality, the top predictors were selected in the following order: cognitive score, MDS-ADL score, bowel incontinence, urinary incontinence, oxygen use, and balance while walking. For predicting successful community discharge, the top predictors were selected in the following order: urinary incontinence, MDS-ADL score, bowel incontinence, balance while walking, cognitive score, and oxygen use. Given that these elements were strongly predictive of both outcomes, we ultimately included them in the final model. We compared the performance of a model using all MDS data elements and the reduced model which showed similar performance.

Measures of added value

We assessed the incremental value of the MDS predictors to the base Medicare model in several ways. First, we compared measures of discrimination (c-statistic) and calibration (calibration plot, calibration slope, calibration intercept, and integrated calibration index) between the base Medicare model and updated MDS model.

Next, we conducted a decision curve analysis by creating a decision curve which quantifies the net benefit across various risk thresholds.^1–4^ Unlike discrimination and calibration, decision curve analysis more directly assesses clinical utility by incorporating the benefits and harms of certain interventions. In the setting of this model, the “decision” may be related to interventions for those at high mortality risk such as comprehensive care planning and case conferences, engaging in more in-depth goals of care discussions, referring to palliative care or hospice, or having a patient undergo a comprehensive medication review to consider deprescribing diabetes and hypertension medications. The benefits of these interventions must be weighed against potential downsides as they may be time and resource intensive.

In a decision curve, the x-axis represents the threshold probability above which a clinician would consider implementing the intervention. A risk threshold of 10% implies a harm-to-benefit ratio of 1:9 (i.e., we would be willing to refer no more than 10 people to palliative care who may or may not die within 6 months in order to find one person who does indeed die within 6 months). Choosing a lower risk threshold means that a clinician is more worried about the outcome (i.e., more willing to accept false positives to ensure that patients who ultimately experience the outcome are not missed). Choosing a higher risk threshold means that a clinician would be more worried about unnecessary interventions following the prediction. The y-axis represents the net benefit, which combines the number of true positives (e.g., referrals to palliative care when the patient dies within 6 months) and false positives (e.g., “unnecessary” referrals to palliative care when the patient does not actually die within 6 months) into a single number. It is measured in terms of the number of true positives and is calculated as [benefit – (harm x exchange rate)], where the exchange rate reflects the balance between the benefit of a true positive and the harm of a false positive (e.g., at a risk threshold of 20%, the harm to benefit ratio is 1:4 and we would weight each false positive by the odds of 5 (20/80)). The net benefit of using the prognostic model is compared against strategies of “Intervene for all” (also referred to as “Treat all”) and “Intervene for none” (also referred to as “Treat none”). The “Intervene for all” line reflects a strategy of having all patients undergo a certain intervention and crosses the x-axis at the prevalence of 6-month mortality or successful community discharge. The “Intervene for none” line reflects a strategy of having no patients undergo a certain intervention. The “Intervene for none” line is equal to 0 since this strategy has no true or false positives. In our decision curve analysis, we compare the updated MDS model, the base Medicare model, a simplified model with only age and sex as predictors, the intervene for all strategy, and intervene for none strategy. A model with higher net benefit across clinically relevant thresholds may show higher clinical utility in supporting clinical decisions. Of note, decision curves cannot be used to choose the best threshold for an intervention. For our analysis, we assessed net benefit across the full range of threshold probabilities from 0-100% given that our model may be used for a variety of interventions. For example, for an intervention of referring patients to palliative care or having a comprehensive care conference based on 6-month mortality risk, we considered a threshold of above ~40% to be clinically relevant.

Finally, we calculated the “fraction of new information provided” to quantify the proportion of total predictive information explained by the additional MDS predictors when added to the *base* Medicare model.^5^ We first calculated the ratio of the likelihood ratio X^2^ value from the base Medicare model to the updated MDS model. We also calculated the ratio of the variance of risk predictions from the base Medicare model to the updated MDS model. One minus these numbers indicates the fraction of new information provided by the MDS predictors (i.e., the proportion of variation that is explained by MDS predictors). We also show a side-by-side histogram of the distribution of risk predictions from the base Medicare model to the updated MDS model and a scatter plot of the individual risk predictions from the base Medicare model and the updated MDS model.

Full equation for the updated MDS 6-month mortality model and example calculation

The full equation for the updated MDS 6-month mortality model is as follows:

logit (Probability of 6-month mortality) = {-5.963+0.021*age+2.74e-05*pmax(age-68,0)^3-1.13e-05*pmax(age-78,0)^3-5.91e-05*pmax(age-85,0)^3+4.30e-05*pmax(age-94,0)^3+0.338*(Male)-0.218*(Medicaid)+0.481*(Weight loss)+0.370*(Heart Failure)+0.032*(Complicated diabetes)+0.524*(Severe renal failure)+1.091*(Severe liver failure)-0.031*(Dementia)-0.027*(Other neurologic disease)+0.198*(Lung disease)-0.245*(Paralysis)+1.791*(Metastatic cancer)+0.326*(Solid cancer)+0.514*(Leukemia/lymphoma)-0.475*(Injury discharge diagnosis)-0.529*(MSK discharge diagnosis)+0.764*(Neoplasm discharge diagnosis)+0.130*(Circulatory discharge diagnosis)+0.535*(Urgent/emergent admission type)+0.0396*(Hospital length of stay)-0.0011*pmax(Hospital LOS-4,0)^3+0.0011*pmax(Hospital LOS-5,0)^3-2.03e-05*pmax(Hospital LOS-8,0)^3-6.28e-05*pmax(Hospital LOS-21,0)^3+0.111*(1 hospitalization in past year)+0.207*(2 hospitalizations in past year)+0.303*(3 hospitalizations in past year)+0.325*(4 or more hospitalizations in the past year)+0.018*(MDS-ADL Score)+0.00023*pmax(MDS-ADL Score-9,0)^3-0.00088*pmax(MDS-ADL Score-17,0)^3+0.00047*pmax(MDS-ADL Score-19,0)^3+0.00018*pmax(MDS-ADL Score-22,0)^3+0.333*(Mild impairment)+0.676*(Moderate impairment)+1.072*(Severe impairment)+0.548*(Oxygen use)+0.104*(Occasionally incontinent of urine)+0.167*(Frequently incontinent of urine)+0.184*(Always incontinent of urine)+0.334*(Urinary incontinence not rated)+0.141*(Occasionally incontinent of bowel)+0.253*(Frequently incontinent of bowel)+0.444*(Always incontinent of bowel)+0.118*(Bowel incontinence not rated)+0.054*(Not steady but stabilizes without assistance)+0.179*(Not steady but stabilizes with assistance)+0.419*(Did not walk) }

As an example, consider an 85-year-old woman discharged to a skilled nursing facility after a hospitalization for a heart failure exacerbation. Her hospital length of stay was 5 days. She does not receive Medicaid and has had 0 hospitalizations in the past year. She has a history of heart failure and hemiplegia following a stroke. Her admission MDS-ADL score is 21, indicating needing extensive assistance with most ADLs. She has mild impairment in cognition based on her admission Cognitive Function Scale assessment. She is not using oxygen on admission to the SNF, is frequently incontinent of bowel and bladder, and did not walk in her first 7 days of SNF admission. After plugging into the formula, her estimated 6-month mortality is 23.7%

Full equation for the updated MDS community discharge model and example calculation

The full equation for the updated MDS 6-month community discharge model is as follows:

logit (Probability of successful community discharge) = {2.020+0.0022*age-3.13e-05*pmax(age-68,0)^3+3.40e-05*pmax(age-78,0)^3+3.00e-05*pmax(age-85,0)^3-3.27e-05*pmax(age-94,0)^3-0.107*(Male)-0.434*(Medicaid)-0.215*(Weight loss)-0.169*(Heart Failure)-0.050*(Complicated diabetes)-0.270*(Severe renal failure)-0.469*(Severe liver failure)-0.182*(Dementia)-0.003*(Other neurologic disease)-0.075*(Lung disease)+0.110*(Paralysis)-0.707*(Metastatic cancer)-0.069*(Solid cancer)-0.223*(Leukemia/lymphoma)+0.288*(Injury discharge diagnosis)+0.374*(MSK discharge diagnosis)-0.330*(Neoplasm discharge diagnosis)-0.052*(Circulatory discharge diagnosis)-0.309*(Urgent/emergent admission type)-0.042*(Hospital LOS)+0.0003*pmax(Hospital LOS-4,0)^3-0.00026*pmax(Hospital LOS-5,0)^3-8.54e-05*pmax(Hospital LOS-8,0)^3+3.52e-05*pmax(Hospital LOS-21,0)^3-0.104*(1 hospitalization in past year)-0.265*(2 hospitalizations in past year)-0.354*(3 hospitalizations in past year)-0.584*(4 or more hospitalizations in the past year)-0.0014*MDS-ADL-0.00026*pmax(MDS-ADL-9,0)^3+0.0011*pmax(MDS-ADL-17,0)^3-0.00075*pmax(MDS-ADL-19,0)^3-0.00012*pmax(MDS-ADL-22,0)^3-0.304*(Mild impairment)-0.577*(Moderate impairment)-0.847*(Severe impairment)-0.241*(Oxygen use)-0.158*(Occasionally incontinent of urine)-0.304*(Frequently incontinent of urine)-0.333*(Always incontinent of urine)-0.476*(Urinary incontinence not rated)-0.111*(Occasionally incontinent of bowel)-0.190*(Frequently incontinent of bowel)-0.371*(Always incontinent of bowel)-0.123*(Bowel incontinence not rated)+0.069*(Not steady but stabilizes without assistance)-0.033*(Not steady but stabilizes with assistance)-0.411*(Did not walk) }

Consider the same 85-year-old woman as above. After plugging into the formula, her estimated probability of successful community discharge is 45.2%. See Table 4 for additional sample calculations.

**Supplementary Figure S1:** Flow diagram for cohort construction

Admitted to a skilled nursing facility within 3 days of hospital discharge from 1/1/2017 – 12/31/2019

(1,277,300 admissions, 860,895 individuals)

Exclude individuals <66 years of age (N=94,606) and people without continuous Medicare FFS coverage (N=161,300)

Age ≥66 years with continuous Medicare A/B FFS coverage during admission

(909,105 admissions, 604,989 individuals)

Exclude people in NH prior to hospitalization using Medicare Part A/B E&M and CPT codes

Not residing in a nursing home in the 3 months prior to SNF admission

(820,138 admissions, 549,090 individuals)

Exclude SNF admissions to US territories (N=207)

Exclude people who were admitted directly to the hospital from SNF/acute rehab/LTAC (N=25,143)

Hospital & SNF admission not directly from SNF/acute rehab/LTAC

(678,740 admissions, 523,740 individuals)

Exclude SNF admissions after first SNF admission

First SNF admission during the study period

(523,740 admissions, 523,740 individuals)

Exclude individuals without MDS records, those who re-entered the facility (rather than new admissions), and those who entered from locations other than hospital

Individuals with MDS data tracking record coded as a new admission from the hospital

(495,443 individuals)

Exclude individuals discharged (either planned or unplanned) or died before day 7

Individuals who remained at a SNF for 7 days

(441,970 individuals)

Exclude individuals without a comprehensive MDS assessment on or before day 7 whose first assessment is not a discharge assessment

Individuals who remained at a SNF for 7 days after admission with an MDS admission assessment

(426, 680 individuals)

**Supplementary Figure S2:** Plot of the proportion of overall$\chi^{2}$ for each predictor in the model when predicting 6-month mortality for older adults admitted to a skilled nursing facility


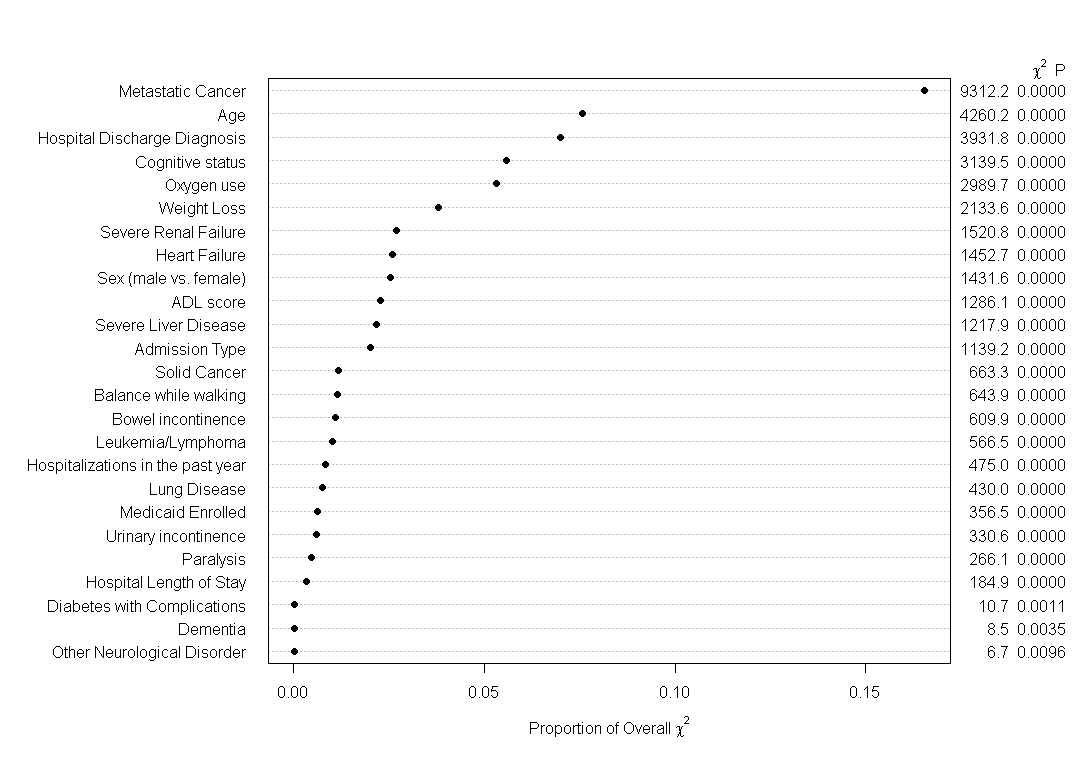


Abbreviations: ADL, activities of daily living

**Supplementary Figure S3:** Plot of the proportion of overall $\chi^{2}$ for each predictor in the model when predicting successful community discharge for older adults admitted to a skilled nursing facility


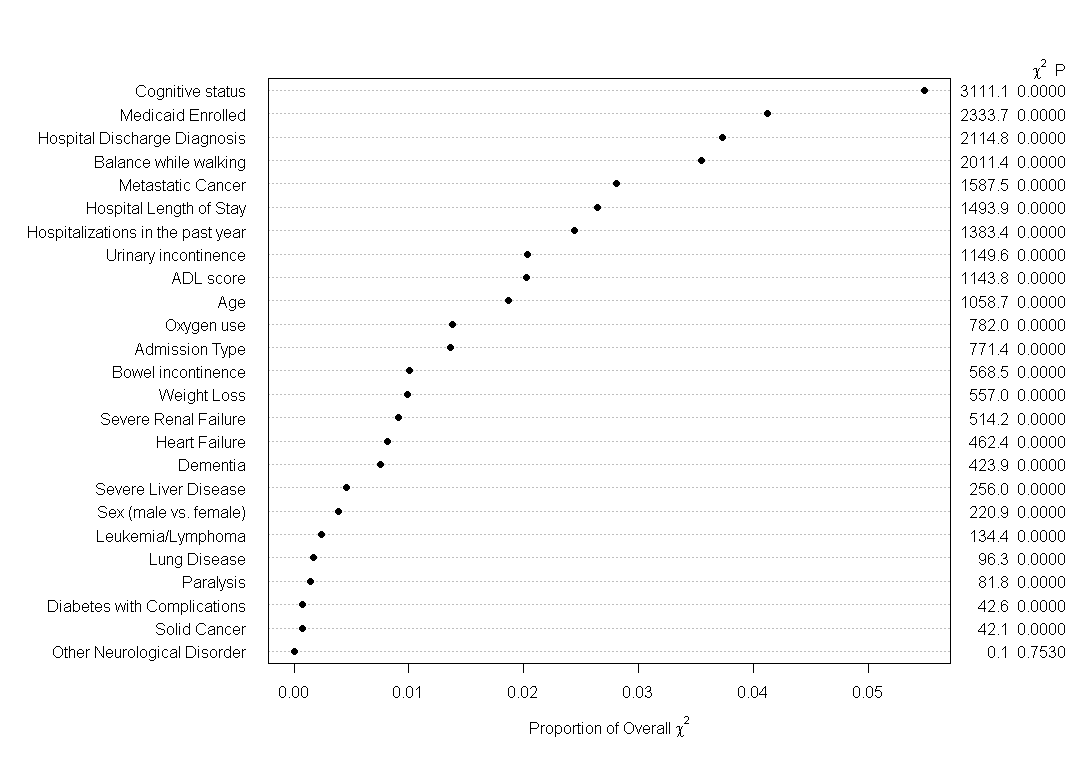


Abbreviations: ADL, activities of daily living

**Supplementary Figure S4**: Calibration plot of the base model in predicting 6-month mortality and successful community discharge


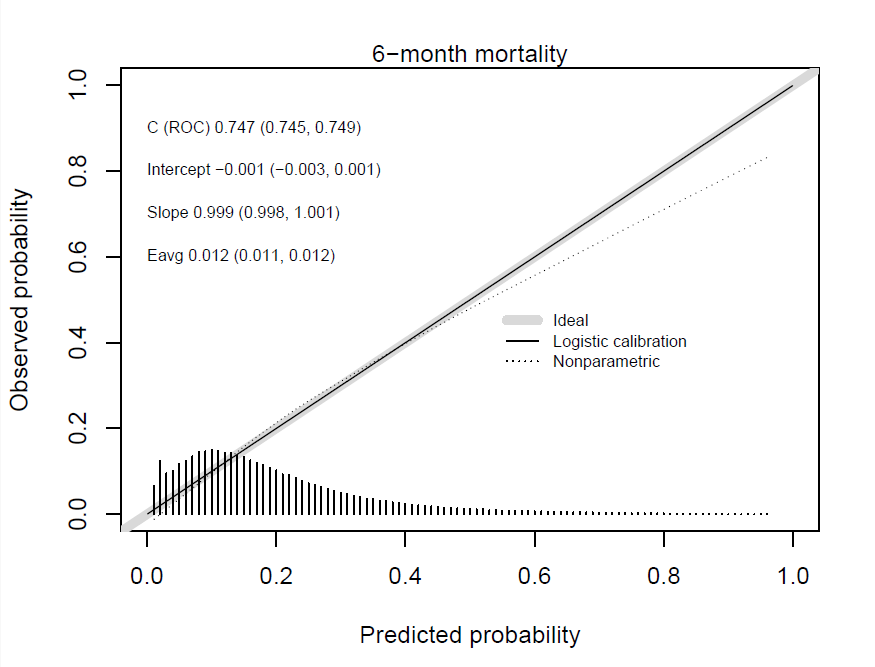


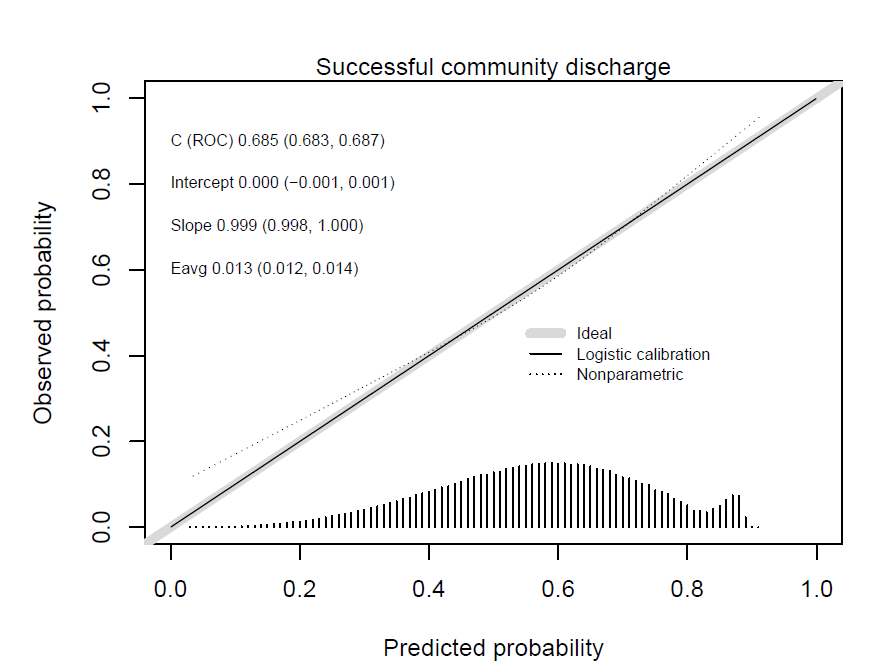


Abbreviations: C (ROC), c-statistic (area under the receiver operating characteristic curve); Eavg, the average absolute difference between observed and predicted probabilities over the range of predicted probabilities

**Supplementary Figure S5:** Decision curve analysis for the outcome of 6-month mortality comparing the net benefit of the base model, updated Minimum Data Set model, a simplified model including only age and sex, and the intervene for all and intervene for none strategies across a range of thresholds


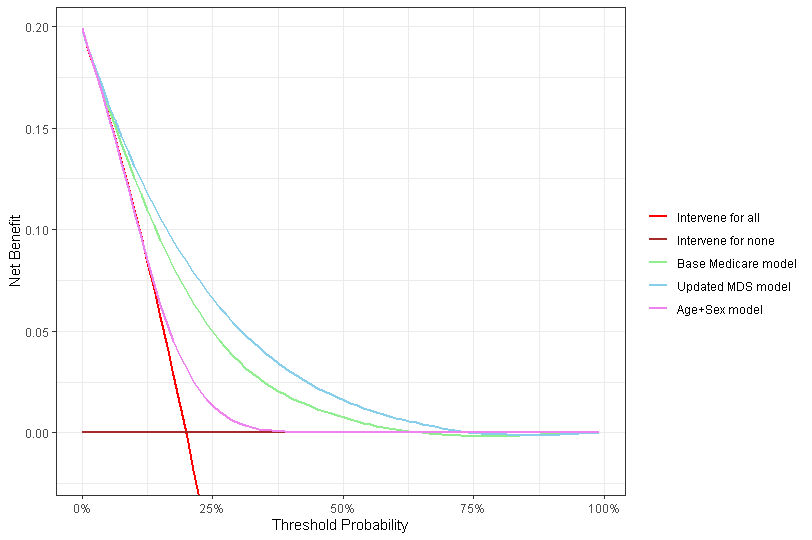


Note: This figure shows the net benefit of using the base Medicare model, updated MDS model, and a simplified model which only includes age and sex as predictors. The unit of net benefit is true positives. To be of clinical value, the net benefit should be higher than zero and higher than the default strategies (intervene for all and intervene for none). The updated MDS model has higher net benefit than the intervene for all, intervene for none, base Medicare model, and a simplified model with only age and sex across a wide range of threshold probabilities from around 15% to 60%. See Supplementary Methods for additional details.

**Supplementary Figure S6:** Decision curve analysis for the outcome of successful community discharge comparing the net benefit of the base model, updated Minimum Data Set model, a simplified model including only age and sex, and the intervene for all and intervene for none strategies across a range of thresholds


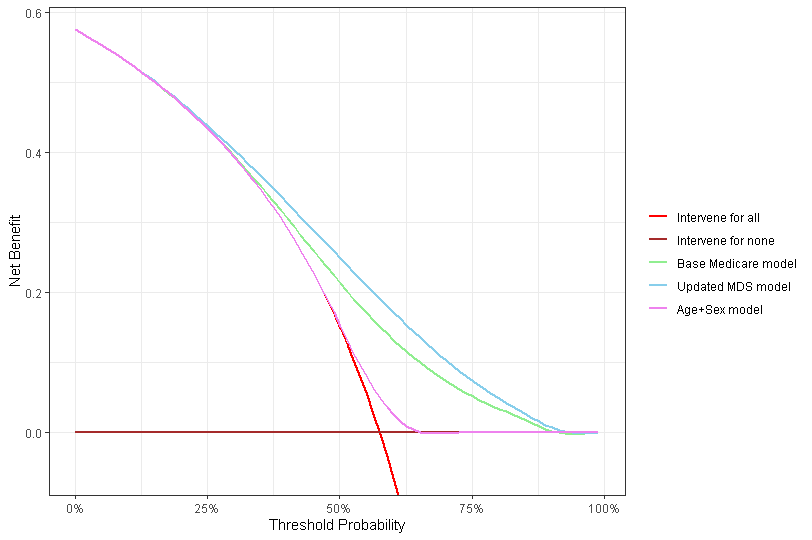


Note: This figure shows the net benefit of using the base Medicare model, updated MDS model, and a simplified model which only includes age and sex as predictors. The unit of net benefit is true positives. To be of clinical value, the net benefit should be higher than zero and higher than the default strategies (intervene for all and intervene for none). The updated MDS model has higher net benefit than the intervene for all, intervene for none, base Medicare model, and a simplified model with only age and sex across a wide range of threshold probabilities from around 35% to 80%. See Supplementary Methods for additional details.

**Supplementary Figure S7:** Back-to-back histogram of predicted risks for the base Medicare model and the updated MDS model when predicting 6-month mortality


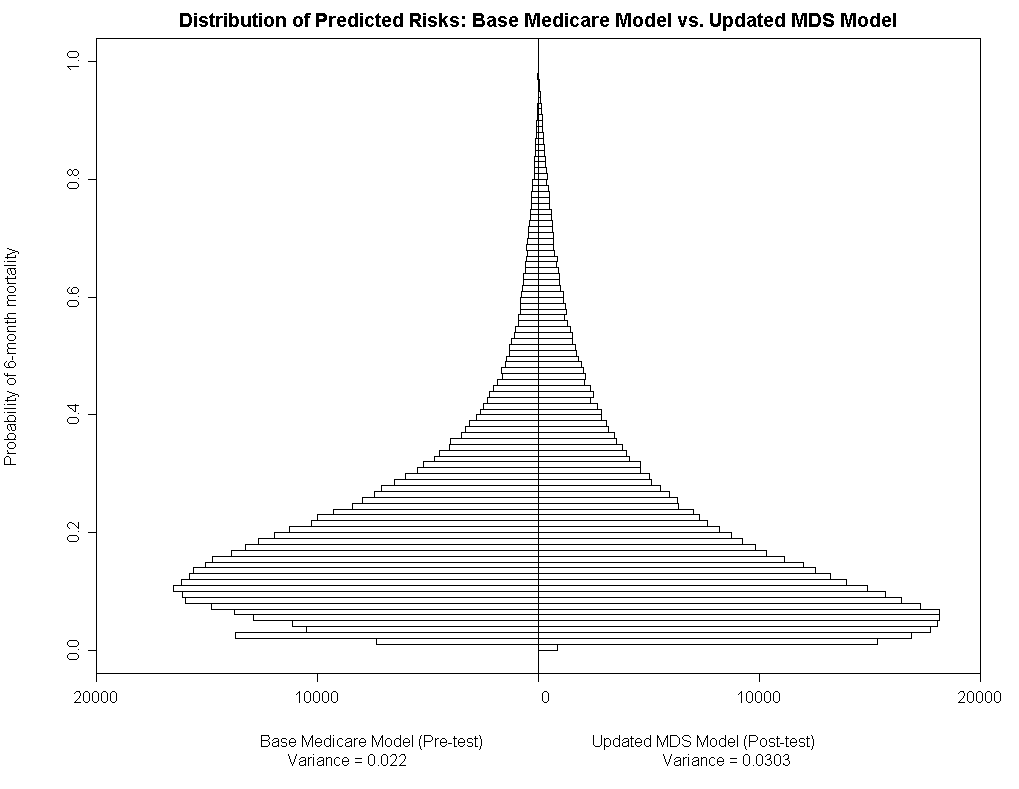


Note: The histograms display the distribution of predicted risk estimates from the base Medicare model (left) and the updated Minimum Data Set model (right). The updated MDS model shows increased variance with a widening of the distribution, indicating greater discrimination.

**Supplementary Figure S8:** Back-to-back histogram of predicted risks for the base Medicare model and the updated MDS model when predicting successful community discharge


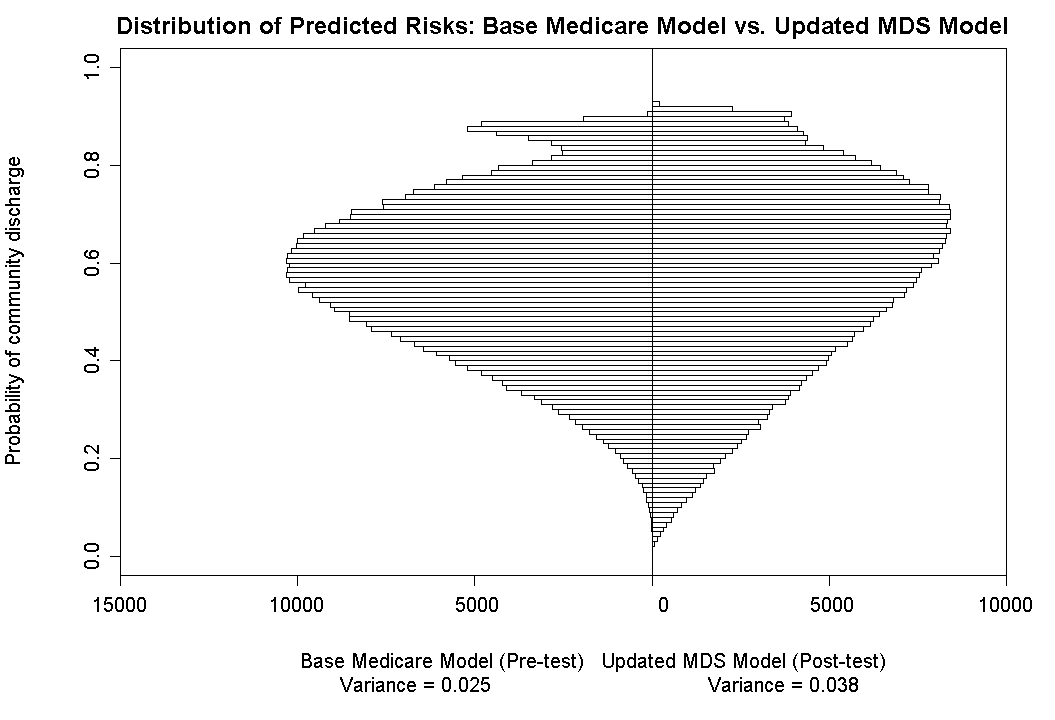


Note: The histograms display the distribution of predicted risk estimates from the base Medicare model (left) and the updated Minimum Data Set model (right). The updated MDS model shows increased variance with a widening of the distribution, indicating greater discrimination.

**Supplementary Figure S9:** Plot of the estimated probability of 6-month mortality for individuals using predictions from the base Medicare model and the updated MDS model


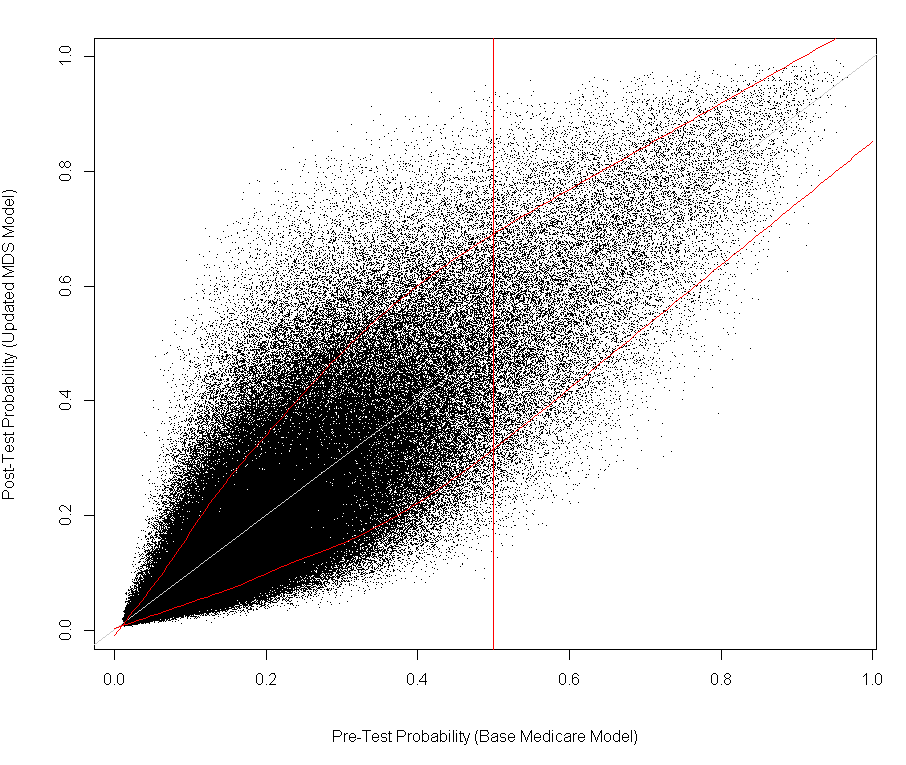


Note: The plot shows the predicted risk estimates for individuals from the base Medicare model (x-axis) against the predicted risk estimates from the updated MDS model (y-axis). The two red lines indicate the 0.1 and 0.9 quantiles of risk estimated from the updated MDS model as a function of risk estimated from the base Medicare model. This suggests that once information from MDS data elements is incorporated into the model, the updated risk of 6-month mortality can vary quite significantly for certain patients.

**Supplementary Figure S10:** Plot of the estimated probability of successful community discharge for individuals using predictions from the base Medicare model and the updated MDS model


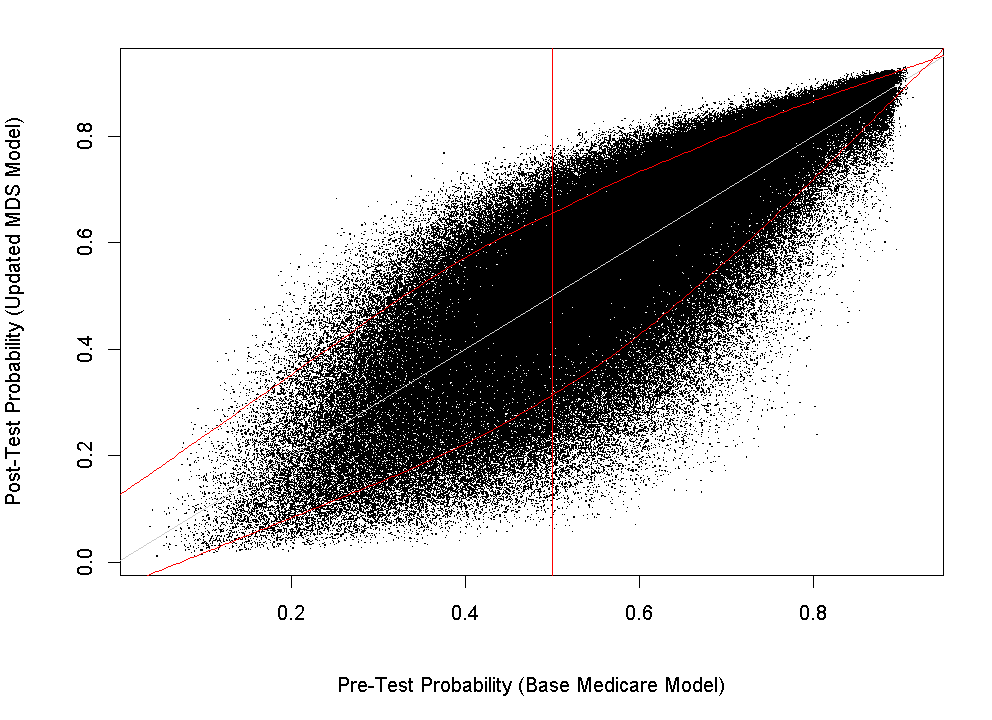


Note: The plot shows the predicted risk estimates for individuals from the base Medicare model (x-axis) against the predicted risk estimates from the updated MDS model (y-axis). The two red lines indicate the 0.1 and 0.9 quantiles of risk estimated from the updated MDS model as a function of risk estimated from the base Medicare model. This suggests that once information from MDS data elements is incorporated into the model, the updated risk of successful community discharge can vary quite significantly for certain patients.

**Supplementary Table S1:** TRIPOD+AI Checklist

| **Section/Topic Item Development Checklist item**  **/ evaluation**^1^ | | | | | | **Reported on page** | | |
| --- | --- | --- | --- | --- | --- | --- | --- | --- |
| **TITLE** | | | | | |  |  |  |
| *Title* | | 1 | D;E | | Identify the study as developing or evaluating the performance of a multivariable prediction model, the target population, and the outcome to be predicted | 1 | | |
| **ABSTRACT** | | | | | | | | |
| *Abstract* | | 2 | D;E | | See TRIPOD+AI for Abstracts checklist | 3 | | |
| **INTRODUCTION** | | | | | | | | |
| *Background* | | 3a | D;E | | Explain the healthcare context (including whether diagnostic or prognostic) and rationale for developing or evaluating the prediction model, including references to existing models | 5 | | |
|  |  | 3b | D;E | | Describe the target population and the intended purpose of the prediction model in the context of the care pathway, including its intended users (e.g., healthcare professionals, patients, public) | 5 | | |
|  |  | 3c | D;E | | Describe any known health inequalities between sociodemographic groups | 5 | | |
| *Objectives* | | 4 | D;E | | Specify the study objectives, including whether the study describes the development or validation of a prediction model (or both) | 5-6 | | |
| **METHODS** | | | | | | | | |
| *Data* | | 5a | D;E | | Describe the sources of data separately for the development and evaluation datasets (e.g., randomised trial, cohort, routine care or registry data), the rationale for using these data, and representativeness of the data | 6 | | |
|  |  | 5b | D;E | | Specify the dates of the collected participant data, including start and end of participant accrual; and, if applicable, end of follow-up | 6 | | |
| *Participants* | | 6a | D;E | | Specify key elements of the study setting (e.g., primary care, secondary care, general population) including the number and location of centres | 6 | | |
|  |  | 6b | D;E | | Describe the eligibility criteria for study participants | 6-7 | | |
|  |  | 6c | D;E | | Give details of any treatments received, and how they were handled during model development or evaluation, if relevant | N/A | | |
| *Data preparation* | | 7 | D;E | | Describe any data pre-processing and quality checking, including whether this was similar across relevant sociodemographic groups | N/A | | |
| *Outcome* | | 8a | D;E | | Clearly define the outcome that is being predicted and the time horizon, including how and when assessed, the rationale for choosing this outcome, and whether the method of outcome assessment is consistent across sociodemographic groups | 7 | | |
|  |  | 8b | D;E | | If outcome assessment requires subjective interpretation, describe the qualifications and demographic characteristics of the outcome assessors | N/A | | |
|  |  | 8c | D;E | | Report any actions to blind assessment of the outcome to be predicted | N/A | | |
| *Predictors* | | 9a | D | | Describe the choice of initial predictors (e.g., literature, previous models, all available predictors) and any pre-selection of predictors before model building | 7-8 | | |
|  |  | 9b | D;E | | Clearly define all predictors, including how and when they were measured (and any actions to blind assessment of predictors for the outcome and other predictors) | 7-8, supplementary tables S2-S4 | | |
|  |  | 9c | D;E | | If predictor measurement requires subjective interpretation, describe the qualifications and demographic characteristics of the predictor assessors | N/A | | |
| *Sample size* | | 10 | D;E | | Explain how the study size was arrived at (separately for development and evaluation), and justify that the study size was sufficient to answer the research question. Include details of any sample size calculation | Supp Methods | | |
| *Missing data* | | 11 | D;E | | Describe how missing data were handled. Provide reasons for omitting any data | 8, supp methods | | |
| *Analytical methods* | | 12a | D | | Describe how the data were used (e.g., for development and evaluation of model performance) in the analysis, including whether the data were partitioned, considering any sample size requirements | 8 | | |
|  |  | 12b | D | | Depending on the type of model, describe how predictors were handled in the analyses (functional form, rescaling, transformation, or any standardisation). | 8, supplementary tables s2-s4 | | |
|  |  | 12c | D | | Specify the type of model, rationale^2^, all model-building steps, including any hyperparameter tuning, and method for internal validation | 8-9, Supp methods | | |
|  |  | 12d | D;E | | Describe if and how any heterogeneity in estimates of model parameter values and model performance was handled and quantified across clusters (e.g., hospitals, countries). See TRIPOD-Cluster for additional considerations^3^ | N/A | | |
|  |  | 12e | D;E | | Specify all measures and plots used (and their rationale) to evaluate model performance (e.g., discrimination, calibration, clinical utility) and, if relevant, to compare multiple models | 8-9 | | |
|  |  | 12f | E | | Describe any model updating (e.g., recalibration) arising from the model evaluation, either overall or for particular sociodemographic groups or settings | N/A | | |
|  |  | 12g | E | | For model evaluation, describe how the model predictions were calculated (e.g., formula, code, object, application programming interface) | 9, Supp methods | | |
| *Class imbalance* | | 13 | D;E | | If class imbalance methods were used, state why and how this was done, and any subsequent methods to recalibrate the model or the model predictions | N/A | | |
| *Fairness* | | 14 | D;E | | Describe any approaches that were used to address model fairness and their rationale | N/A | | |
| *Model output* | | 15 | D | | Specify the output of the prediction model (e.g., probabilities, classification). Provide details and rationale for any classification and how the thresholds were identified | 9, supp methods | | |
| *Training versus*  *evaluation* | | 16 | D;E | | Identify any differences between the development and evaluation data in healthcare setting, eligibility criteria, outcome, and predictors | N/A | | |
| *Ethical approval* | | 17 | D;E | | Name the institutional research board or ethics committee that approved the study and describe the participant-informed consent or the ethics committee waiver of informed consent | 9 | | |
| **OPEN SCIENCE** | | | | | | | | |
| *Funding* | | 18a | | D;E | Give the source of funding and the role of the funders for the present study | | | See acknowledgments section |
| *Conflicts of interest* | | 18b | | D;E | Declare any conflicts of interest and financial disclosures for all authors | | | See acknowledgments section |
| *Protocol* | | 18c | | D;E | Indicate where the study protocol can be accessed or state that a protocol was not prepared | | | N/A |
| *Registration* | | 18d | | D;E | Provide registration information for the study, including register name and registration number, or state that the study was not registered | | | N/A |
| *Data sharing* | | 18e | | D;E | Provide details of the availability of the study data | | | N/A |
| *Code sharing* | | 18f | | D;E | Provide details of the availability of the analytical code^4^ | | | N/A |
| **PATIENT & PUBLIC INVOLVEMENT** | | | | | | | | |
| *Patient & Public Involvement* | | 19 | | D;E | Provide details of any patient and public involvement during the design, conduct, reporting, interpretation, or dissemination of the study or state no involvement. | | | N/A |
| **RESULTS** | | | | | | | | |
| *Participants* | | 20a | | D;E | Describe the flow of participants through the study, including the number of participants with and without the outcome and, if applicable, a summary of the follow-up time. A diagram may be helpful. | | | 9, supp figure s1 |
|  |  | 20b | | D;E | Report the characteristics overall and, where applicable, for each data source or setting, including the key dates, key predictors (including demographics), treatments received, sample size, number of outcome events, follow-up time, and amount of missing data. A table may be helpful. Report any differences across key demographic groups. | | | 9-10, table 1, supp table s5 |
|  |  | 20c | | E | For model evaluation, show a comparison with the development data of the distribution of important predictors (demographics, predictors, and outcome). | | | N/A |
| *Model development* | | 21 | | D;E | Specify the number of participants and outcome events in each analysis (e.g., for model development, hyperparameter tuning, model evaluation) | | | Table 1 |
| *Model specification* | | 22 | | D | Provide details of the full prediction model (e.g., formula, code, object, application programming interface) to allow predictions in new individuals and to enable third-party evaluation and implementation, including any restrictions to access or re-use (e.g., freely available, proprietary)^5^ | | | Supp methods, supp table s8-s9 |
| *Model performance* | | 23a | | D;E | Report model performance estimates with confidence intervals, including for any key subgroups (e.g., sociodemographic). Consider plots to aid presentation. | | | Table 3, supp table s10, supp tables s12-13 |
|  |  | 23b | | D;E | If examined, report results of any heterogeneity in model performance across clusters. See TRIPOD Cluster for additional details^3^. | | | N/A |
| *Model updating* | | 24 | | E | Report the results from any model updating, including the updated model and subsequent performance | | | N/A |
| **DISCUSSION** | | | | | | | | |
| *Interpretation* | | 25 | | D;E | Give an overall interpretation of the main results, including issues of fairness in the context of the objectives and previous studies | | | 12 |
| *Limitations* | | 26 | | D;E | Discuss any limitations of the study (such as a non-representative sample, sample size, overfitting, missing data) and their effects on any biases, statistical uncertainty, and generalizability | | | 14-15 |
| *Usability of the model in the context of current care* | | 27a | | D | Describe how poor quality or unavailable input data (e.g., predictor values) should be assessed and handled when implementing the prediction model | | | N/A |
|  |  | 27b | | D | Specify whether users will be required to interact in the handling of the input data or use of the model, and what level of expertise is required of users | | | 14 |
|  |  | 27c | | D;E | Discuss any next steps for future research, with a specific view to applicability and generalizability of the model | | | 15 |

**Supplementary Table S2:**Variables included in the base model, including specification

| **Variable** | **Description** | **Scale** | **Specification** |
| --- | --- | --- | --- |
| **Demographics** | | | |
| Age | Age in years at the time of index skilled nursing facility admission | Continuous | 4-knot restricted cubic spline |
| Sex | Biological sex | Categorical | Male vs. female |
| Medicaid enrolled | Enrolled in Medicaid | Categorical | Yes vs. no |
| **Individual comorbidities** | | | |
| Weight loss | Includes diagnosis codes for protein-calorie malnutrition | Categorical | Yes vs. no |
| Heart failure | Includes diagnosis codes for heart failure (e.g., systolic heart failure, diastolic heart failure) | Categorical | Yes vs. no |
| Diabetes (complicated) | Includes diagnosis codes for diabetes with complications (e.g., nephropathy, retinopathy, ulcer) | Categorical | Yes vs. no |
| Renal failure (severe) | Includes diagnosis codes for chronic kidney disease stage 4, chronic kidney disease stage 5 and end stage renal disease | Categorical | Yes vs. no |
| Liver failure (severe) | Includes diagnosis codes for severe liver disease (e.g., cirrhosis with complications such as esophageal varices) | Categorical | Yes vs. no |
| Dementia | Includes diagnosis codes for dementia (e.g., Alzheimer’s disease, vascular dementia) | Categorical | Yes vs. no |
| Other neurologic disease | Includes diagnosis codes for neurologic diseases such as multiple sclerosis, hydrocephalus, hepatic encephalopathy | Categorical | Yes vs. no |
| Lung disease | Includes a combination of diagnosis codes for chronic lung diseases (e.g., asthma, chronic obstructive pulmonary disease, chronic bronchitis) and pulmonary circulatory diseases (e.g., pulmonary hypertension). This category combines the “cmr_lung_chronic” and “cmr_pulmcirc” Elixhauser variables. | Categorical | Yes vs. no |
| Metastatic cancer | Includes diagnosis codes for metastatic cancer (e.g., brain, bladder, kidney, ovary) | Categorical | Yes vs. no |
| Solid cancer | Includes diagnosis codes for solid cancers (e.g., gastrointestinal, lung, kidney, prostate) | Categorical | Yes vs. no |
| Leukemia/lymphoma | Includes a combination of diagnosis codes for leukemia (e.g., acute lymphoblastic leukemia, chronic lymphocytic leukemia) and lymphoma (e.g., Hodgkin lymphoma, follicular lymphoma). This category combines the “cmr_cancer_leuk” and “cmr_cancer_lymph” Elixhauser variables. | Categorical | Yes vs. no |
| Paralysis | Includes diagnosis codes for paraplegia, hemiplegia, quadriplegia | Categorical | Yes vs. no |
| **Hospitalization-related factors** | | | |
| Admission type | Indicates whether the hospitalization was consider emergent/urgent vs. elective | Categorical | Emergent/urgent vs. elective |
| Length of stay | Index hospital length of stay in days (truncated at 30 days) | Continuous | 4-knot restricted cubic spline |
| Principal hospital discharge diagnosis | First diagnosis code from the Medicare discharge claim corresponding to index hospitalization (DGNS_1_CD) based on CCSR groupings | Categorical | Each individual is assigned to a single category out of the 5 categories of discharge diagnoses shown in Supplementary Table S3 (injury, musculoskeletal, circulatory, neoplasm, other) |
| **Prior healthcare utilization** | | | |
| Hospitalizations in last year | Number of acute care hospitalizations in the 365 days prior to index hospitalization | Categorical | 0, 1, 2, 3, 4 or more hospitalizations |

Abbreviations: CCSR, Clinical Classifications Software Refined

**Supplementary Table S3:** Categorization of principal hospital discharge diagnoses according to the Clinical Classifications Software Refined (CCSR) for ICD-10 diagnoses

| **Categories** | **Abbreviation** | **Number** **(%)** |
| --- | --- | --- |
| Other diagnoses^a^ | OTH | 184,886 (43.3%) |
| Injury, poisoning, and certain other consequences of external causes (e.g., fractures) | INJ | 100,350 (23.5%) |
| Diseases of the circulatory system (e.g., heart failure, stroke) | CIR | 76,340 (17.9%) |
| Diseases of the musculoskeletal system and connective tissue (e.g., knee and hip replacement) | MUS | 52,411 (12.3%) |
| Neoplasms (e.g., admissions for chemotherapy) | NEO | 12,693 (3.0%) |

a The “Other” category includes certain infectious and parasitic diseases, diseases of the respiratory system, diseases of the genitourinary system, diseases of the digestive system, diseases of the nervous system, endocrine/nutritional/metabolic diseases, symptoms/signs/abnormal clinical and laboratory findings, diseases of the skin and subcutaneous tissue, diseases of the blood and blood-forming organs and certain disorders involving the immune mechanism, dental diseases, diseases of the ear and mastoid process, external causes of morbidity, diseases of the eye and adnexa, factors influencing health status and contact with health services, congenital malformations, deformations, and chromosomal abnormalities, mental, behavioral, and neurodevelopment disorders, certain conditions originating in the perinatal period, pregnancy, childbirth, and the puerperium, and unacceptable principal diagnosis.

**Supplementary Table S4:** Candidate predictor variables considered for inclusion in the updated Minimum Data Set model, including specification

| **Predictor** | **Description** | **Specification** | **Final variable** | **Parameters (degrees of freedom)** |
| --- | --- | --- | --- | --- |
| Marital status | Identifies the individual’s marital status | Binary variable indicating married vs. never married or widowed or separated or divorced | 1 = Married  2 = Not married | 1 |
| Ability to express ideas or understand others | Ability to express ideas and wants and/or understanding verbal content | Binary variable indicating sometimes/rarely understood or sometimes/rarely understands vs. understood/understands or usually understood/understands | 1 = Does not understand or is not understood  2 = Understands or is understood | 1 |
| Cognitive function scale | Cognitive screen based on the Brief Interview for Mental Status (BIMS) and/or Cognitive Performance Scale (CPS). The BIMS screener is scored from 0-15 and includes 5 items: three questions assessing temporal orientation and two questions assessing recall. The CPS is scored from 0-6 and is based on five MDS items. | 4 level variable with categories of: cognitively intact (BIMS score 13-15), mildly impaired (BIMS score of 8-12 or CPS score of 0-2), moderately impaired (BIMS score of 0-7 or CPS score of 3-4), and severely impaired (CPS score of 5-6) | 1 = Cognitively intact  2 = Mild impairment  3 = Mod impairment  4 = Sev impairment | 3 |
| Delirium | Indication of delirium by the Confusion Assessment Method (item A = acute mental status change; item B = inattention; item C = disorganized thinking; item D = altered level of consciousness) | Yes vs. No  Yes = (Item A = 1 OR Item B, C or D = 2) AND (Item B = 1 OR 2) AND EITHER  (Item C = 1 OR 2 OR Item D = 1 OR 2).  Note: for items B, C, and D, 1 = behavior continuously present, does not fluctuate; 2 = behavior present and fluctuates | 0 = No delirium  1 = Delirium | 1 |
| Mood | Resident mood interview (PHQ-9) or Staff assessment of resident mood (PHQ-9-OV) | Binary variable with score of 0-9 on either the PHQ-9 or PHQ-9-OV indicating no depression vs. greater than/equal to 10 on the PHQ-9 or PHQ-9-OV indicating depression | 0 = No depression  1 = Depression | 1 |
| Psychosis | Potential indicators of psychosis (hallucinations, delusions) | Binary variable indicating presence of hallucinations/delusions vs. none | 0 = No psychosis  1 = Psychosis | 1 |
| Agitated and reactive behavior scale (ARBS) | Composite score involving questions related to physical and verbal behavioral symptoms, other behavioral symptoms, and rejection of care | Score ranges from 0 (behavior not exhibited on all four items) to 12 (behavior occurred daily on all four items). Given the high prevalence of 0 scores, we categorized this as 0 vs. >0. | 0 = No agitation  1 = Agitation | 1 |
| Functional status (MDS-ADL long form score) | Composite score assessing ADLs of bed mobility, transfer, locomotion on unit, dressing, eating, toileting, and personal hygiene. | Continuous score with range of 0-28  Each ADL is scored as either 0 (independent), 1 (supervision), 2 (limited assistance), 3 (extensive assistance), and 4 (total dependence). For each ADL, we recoded any items with scores of 7 or 8 (activity occurred only once or twice or activity did not occur) as totally dependent, code 4. | Continuous score of 0-28 modeled as RCS with 4 knots | 3 |
| Bathing | How resident takes full-body bath/shower, sponge bath, and transfers in/out of tub/shower (excludes washing of back and hair) | Binary variable indicating needs assistance (codes 2, 3, 4, 8) vs. independent/supervision (codes 0,1) | 0 = Needs assistance  1 = Independent | 1 |
| Balance while walking | Resident's balance while walking (with assistive device if used). | 0=Steady at all times  1=Not steady, but able to stabilize without human assistance  2=Not steady, only able to stabilize with human assistance  8=Activity did not occur | 0 = Steady at all time  1 = Not steady but stabilizes without assistance  2 = Not steady, stabilizes with assistance  8 = Did not walk | 3 |
| Urinary catheter | Presence of indwelling catheter (including suprapubic catheter and nephrostomy tube) | Yes vs. No | 0 = No catheter  1 = Catheter | 1 |
| Ostomy | Presence of ostomy (urostomy, ileostomy, colostomy) | Yes vs. No | 0 = No ostomy  1 = Ostomy | 1 |
| Urinary incontinence | Assessment of urinary incontinence | Always continent vs. occasionally incontinent vs. frequently incontinence vs. always incontinent vs. not rated | 0 = Always continent  1 = Occasionally incontinent  2 = Frequently incontinent  3 = Always incontinent  9 = Not rated (e.g., urinary catheter) | 4 |
| Bowel incontinence | Assessment of bowel incontinence | Always continent vs. occasionally incontinent vs. frequently incontinence vs. always incontinent vs. not rated | 0 = Always continent  1 = Occasionally incontinent  2 = Frequently incontinent  3 = Always incontinent  9 = Not rated (e.g., ostomy) | 4 |
| Any shortness of breath | Shortness of breath with exertion, when sitting at rest, or when lying flat | Binary variable with any shortness of breath vs. none | 0 = Any shortness of breath  1 = No shortness of breath | 1 |
| Fever | Fever | Yes vs no | 0 = No fever  1 = Fever | 1 |
| Vomiting | Vomiting | Yes vs no | 0 = No vomiting  1 = Vomiting | 1 |
| Dehydrated | Dehydrated | Yes vs no | 0 = Dehydrated  1 = Not dehydrated | 1 |
| Internal bleeding | Internal bleeding | Yes vs no | 0 = Internal bleeding  1 = No bleeding | 1 |
| Fall history | Fall in the last 6 months | Binary variable indicating fall in last 30 days or 2-6 months vs. no fall or not assessed | 0 = No fall  1 = Fall | 1 |
| Any swallowing disorder | Signs and symptoms of possible swallowing disorder | Binary variable indicating any issues with swallowing (choking, holding food, lost liquid/solid, or difficulty/pain with swallowing) vs. none of the above | 0 = Trouble swallowing  1 = No trouble swallowing | 1 |
| Body mass index | Body mass index | Continuous variable. BMI = Weight / height2. Set height of >84 and <48 inches to missing. Set weight of <70 pounds and 999 pounds to missing. | Continuous | 1 |
| Weight loss | Loss of 5% or more in the last month or loss of 10% or more in last 6 months | Yes (yes, on physician prescribed weight-gain regimen or yes, not on physician-prescribed weight-gain regimen) vs. no | 0 = No weight loss  1 = Weight loss | 1 |
| Nutritional approaches | Indicates parenteral/intravenous feeding or feeding tube | Parenteral/intravenous feeding or feeding tube vs. no | 0 = No feeding tube or total parental nutrition  1 = Feeding tube or total parental nutrition | 1 |
| Pressure ulcer | At least one stage 3, 4, or unstageable pressure ulcer | At least one stage 3, 4, or unstageable pressure ulcer vs. none | 0 = No pressure ulcer  1 = Pressure ulcer | 1 |
| Oxygen therapy | Oxygen therapy | Binary variable indicating oxygen therapy while a resident of the facility vs. no | 0 = No oxygen use  1 = Oxygen use | 1 |
| Tracheostomy or mechanical ventilation | Tracheostomy care or invasive mechanical ventilator | Binary variable indicating tracheostomy care or invasive mechanical ventilator while a resident or within last 14 days while not a resident of the facility vs. no | 0 = No trach/vent use  1 = Trach/vent use | 1 |
| Intravenous medication | Intravenous medication use | Binary variable indicating intravenous medication while a resident vs. no | 0 = No IV med  1 = IV meds | 1 |
| Dialysis | Dialysis | Binary variable indicating dialysis while a resident of the facility vs. no | 0 = No dialysis  1 = Dialysis | 1 |
| Chemotherapy or radiation therapy | Chemotherapy or radiation | Binary variable indicating chemotherapy or radiation while a resident of the facility vs. no | 0 = No chemo/radiation  1 = Chemo/radiation | 1 |
| Pain | Pain | Binary variable indicating whether the resident has had pain or hurting in the last five days | 0 = No pain  1 = Pain | 1 |
| Anticoagulant use | Any use of anticoagulant in the prior 7 days | Binary variable indicating whether the resident received an anticoagulant in the last 7 days  >0 days vs. 0 days | 0 = No anticoagulant  1 = Anticoagulant | 1 |
| Antipsychotic use | Any use of antipsychotic in the prior 7 days | Binary variable indicating whether the resident received an antipsychotic in the last 7 days  >0 days vs. 0 days | 0 = No antipsychotic  1 = Antipsychotic | 1 |
| Hypnotic use | Any use of hypnotic in the prior 7 days | Binary variable indicating whether the resident received a hypnotic in the last 7 days  >0 days vs. 0 days | 0 = No hypnotic  1 = Hypnotic | 1 |
| Diuretic use | Any use of diuretic in the prior 7 days | Binary variable indicating whether the resident received a diuretic in the last 7 days  >0 days vs. 0 days | 0 = No diuretic  1 = Diuretic | 1 |

**Supplementary Table S5:** Baseline characteristics of individuals admitted to a skilled nursing facility for post-acute care from 2017-2019 who remained at a skilled nursing facility on day 7 and had a completed Minimum Data Set admission assessment

|  |  | **6 month Mortality Outcome** | | **Successful Community Discharge Outcome** | |
| --- | --- | --- | --- | --- | --- |
| **Characteristic** | **Overall (N=426,680)** | **Alive at 6 months (N=341,966)** | **Dead at 6 months (N=84,714)** | **Successful community discharge (N=245,591)** | **Did not experience a successful community discharge (N=181,189)** |
| Age in years, mean (SD) | 81.3 (8.26) | 80.8 (8.17) | 83.2 (8.35) | 80.8 (8.11) | 82.0 (8.42) |
| Age category, years |  |  |  |  |  |
| 66-70 | 52624 (12.3%) | 45087 (13.2%) | 7537 (8.9%) | 31773 (12.9%) | 20851 (11.5%) |
| 71-75 | 64926 (15.2%) | 54983 (16.1%) | 9943 (11.7%) | 39897 (16.3%) | 25029 (13.8%) |
| 76-80 | 77660 (18.2%) | 64188 (18.8%) | 13472 (15.9%) | 46964 (19.1%) | 30696 (16.9%) |
| 81-85 | 86198 (20.2%) | 69322 (20.3%) | 16876 (19.9%) | 50264 (20.5%) | 35934 (19.8%) |
| 88-90 | 82372 (19.3%) | 63574 (18.6%) | 18798 (22.2%) | 45275 (18.4%) | 37097 (20.5%) |
| 90+ | 62900 (14.7%) | 44812 (13.1%) | 18088 (21.4%) | 31318 (12.8%) | 31582 (17.4%) |
| Female sex | 267389 (62.7%) | 221659 (64.8%) | 45730 (54.0%) | 159435 (64.9%) | 107954 (59.6%) |
| Race/Ethnicity |  |  |  |  |  |
| Non-Hispanic White | 362591 (85.0%) | 290243 (84.9%) | 72348 (85.4%) | 210809 (85.9%) | 151782 (83.8%) |
| Non-Hispanic Black | 33502 (7.9%) | 26625 (7.8%) | 6877 (8.1%) | 17444 (7.1%) | 16058 (8.9%) |
| Hispanic | 16173 (3.8%) | 13208 (3.9%) | 2965 (3.5%) | 8923 (3.6%) | 7250 (4.0%) |
| Asian/Pacific Islander | 7856 (1.8%) | 6406 (1.9%) | 1450 (1.7%) | 4398 (1.8%) | 3458 (1.9%) |
| Native American | 1868 (0.4%) | 1527 (0.4%) | 341 (0.4%) | 1039 (0.4%) | 829 (0.5%) |
| Other | 2530 (0.6%) | 2117 (0.6%) | 413 (0.5%) | 1518 (0.6%) | 1012 (0.6%) |
| Unknown | 2160 (0.5%) | 1840 (0.5%) | 320 (0.4%) | 1360 (0.6%) | 800 (0.4%) |
| Medicaid status |  |  |  |  |  |
| Yes | 76183 (17.9%) | 61224 (17.9%) | 14959 (17.7%) | 34518 (14.1%) | 41665 (23.0%) |
| No | 350497 (82.1%) | 280742 (82.1%) | 69755 (82.3%) | 210973 (85.9%) | 139524 (77.0%) |
| Individual comorbidities |  |  |  |  |  |
| Weight loss | 74712 (17.5%) | 50227 (14.7%) | 24485 (28.9%) | 33532 (13.7%) | 41180 (22.7%) |
| Heart failure | 154437 (36.2%) | 112975 (33.0%) | 41462 (48.9%) | 78231 (31.9%) | 76206 (42.1%) |
| Diabetes (complicated) | 130844 (30.7%) | 102828 (30.1%) | 28016 (33.1%) | 70706 (28.8%) | 60138 (33.2%) |
| Renal failure (severe) | 39361 (9.2%) | 26766 (7.8%) | 12595 (14.9%) | 17797 (7.2%) | 21564 (11.9%) |
| Liver failure (severe) | 5644 (1.3%) | 3391 (1.0%) | 2253 (2.7%) | 2418 (1.0%) | 3226 (1.8%) |
| Dementia | 118818 (27.8%) | 89184 (26.1%) | 29634 (35.0%) | 54458 (22.2%) | 64360 (35.5%) |
| Other neurologic disease | 92971 (21.8%) | 69221 (20.2%) | 23750 (28.0%) | 43984 (17.9%) | 48987 (27.0%) |
| Lung disease | 164566 (38.6%) | 125741 (36.8%) | 38825 (45.8%) | 89626 (36.5%) | 74940 (41.4%) |
| Paralysis | 41327 (9.7%) | 32342 (9.5%) | 8985 (10.6%) | 20140 (8.2%) | 21187 (11.7%) |
| Metastatic cancer | 18928 (4.4%) | 8315 (2.4%) | 10613 (12.5%) | 7296 (3.0%) | 11632 (6.4%) |
| Solid cancer | 52132 (12.2%) | 39361 (11.5%) | 12771 (15.1%) | 29216 (11.9%) | 22916 (12.6%) |
| Leukemia/lymphoma | 13258 (3.1%) | 8881 (2.6%) | 4377 (5.2%) | 6610 (2.7%) | 6648 (3.7%) |
| Hospital length of stay, mean (SD) | 8.20 (5.68) | 7.93 (5.50) | 9.31 (6.21) | 7.48 (5.00) | 9.18 (6.35) |
| Admission type |  |  |  |  |  |
| Urgent/emergent | 355725 (83.4%) | 277999 (81.3%) | 77726 (91.8%) | 194840 (79.4%) | 160885 (88.8%) |
| Elective | 70955 (16.6%) | 63967 (18.7%) | 6988 (8.2%) | 50651 (20.6%) | 20304 (11.2%) |
| Principal discharge diagnosis group |  |  |  |  |  |
| Injury (e.g., fracture) | 100350 (23.5%) | 86521 (25.3%) | 13829 (16.3%) | 62765 (25.6%) | 37585 (20.7%) |
| Circulatory (e.g., heart failure, stroke) | 76340 (17.9%) | 58023 (17.0%) | 18317 (21.6%) | 40339 (16.4%) | 36001 (19.9%) |
| Musculoskeletal (e.g., knee replacement) | 52411 (12.3%) | 48925 (14.3%) | 3486 (4.1%) | 40748 (16.6%) | 11663 (6.4%) |
| Neoplastic | 12693 (3.0%) | 6765 (2.0%) | 5928 (7.0%) | 5531 (2.3%) | 7162 (4.0%) |
| Other | 184886 (43.3%) | 141732 (41.4%) | 43154 (50.9%) | 96108 (39.1%) | 88778 (49.0%) |
| Hospitalizations in the past year |  |  |  |  |  |
| 0 | 264981 (62.1%) | 219772 (64.3%) | 45209 (53.4%) | 162508 (66.2%) | 102473 (56.6%) |
| 1 | 93887 (22.0%) | 73320 (21.4%) | 20567 (24.3%) | 51847 (21.1%) | 42040 (23.2%) |
| 2 | 36661 (8.6%) | 27194 (8.0%) | 9467 (11.2%) | 18114 (7.4%) | 18547 (10.2%) |
| 3 | 16038 (3.8%) | 11379 (3.3%) | 4659 (5.5%) | 7294 (3.0%) | 8744 (4.8%) |
| 4+ | 15113 (3.5%) | 10301 (3.0%) | 4812 (5.7%) | 5728 (2.3%) | 9385 (5.2%) |
| ADL score, mean (SD) (range 0-28)^b^ | 17.0 (3.99) | 16.7 (3.99) | 18.4 (3.69) | 16.3 (3.96) | 18.0 (3.83) |
| Cognitive status |  |  |  |  |  |
| No impairment | 264125 (61.9%) | 225081 (65.8%) | 39044 (46.1%) | 173149 (70.5%) | 90976 (50.2%) |
| Mild impairment | 89581 (21.0%) | 68461 (20.0%) | 21120 (24.9%) | 45546 (18.6%) | 44035 (24.3%) |
| Moderate impairment | 61315 (14.4%) | 41857 (12.2%) | 19458 (23.0%) | 23404 (9.5%) | 37911 (20.9%) |
| Severe impairment | 8452 (2.0%) | 4369 (1.3%) | 4083 (4.8%) | 1913 (0.8%) | 6539 (3.6%) |
| Missing | 3207 (0.8%) | 2198 (0.6%) | 1009 (1.2%) | 1479 (0.6%) | 1728 (1.0%) |
| Oxygen use |  |  |  |  |  |
| Yes | 94386 (22.1%) | 65284 (19.1%) | 29102 (34.4%) | 45906 (18.7%) | 48480 (26.8%) |
| No | 332160 (77.8%) | 276569 (80.9%) | 55591 (65.6%) | 199506 (81.3%) | 132654 (73.2%) |
| Missing | 134 (0.0%) | 113 (0.0%) | 21 (0.0%) | 79 (0.0%) | 55 (0.0%) |
| Urinary incontinence |  |  |  |  |  |
| Always continent | 147524 (34.6%) | 129623 (37.9%) | 17901 (21.1%) | 104285 (42.5%) | 43239 (23.9%) |
| Occasionally incontinent | 122524 (28.7%) | 100891 (29.5%) | 21633 (25.5%) | 74477 (30.3%) | 48047 (26.5%) |
| Frequently incontinent | 95561 (22.4%) | 71380 (20.9%) | 24181 (28.5%) | 44613 (18.2%) | 50948 (28.1%) |
| Always incontinent | 29929 (7.0%) | 18892 (5.5%) | 11037 (13.0%) | 9320 (3.8%) | 20609 (11.4%) |
| Not rated (e.g., urinary catheter) | 31034 (7.3%) | 21104 (6.2%) | 9930 (11.7%) | 12754 (5.2%) | 18280 (10.1%) |
| Missing | 108 (0.0%) | 76 (0.0%) | 32 (0.0%) | 42 (0.0%) | 66 (0.0%) |
| Bowel incontinence |  |  |  |  |  |
| Always continent | 230044 (53.9%) | 198438 (58.0%) | 31606 (37.3%) | 154668 (63.0%) | 75376 (41.6%) |
| Occasionally incontinent | 57019 (13.4%) | 45523 (13.3%) | 11496 (13.6%) | 32136 (13.1%) | 24883 (13.7%) |
| Frequently incontinent | 89989 (21.1%) | 66244 (19.4%) | 23745 (28.0%) | 41805 (17.0%) | 48184 (26.6%) |
| Always incontinent | 40478 (9.5%) | 24878 (7.3%) | 15600 (18.4%) | 12215 (5.0%) | 28263 (15.6%) |
| Not rated (e.g., ostomy) | 8962 (2.1%) | 6746 (2.0%) | 2216 (2.6%) | 4570 (1.9%) | 4392 (2.4%) |
| Missing | 188 (0.0%) | 137 (0.0%) | 51 (0.1%) | 97 (0.0%) | 91 (0.1%) |
| Balance while walking |  |  |  |  |  |
| Steady at all times | 13083 (3.1%) | 11710 (3.4%) | 1373 (1.6%) | 9125 (3.7%) | 3958 (2.2%) |
| Not steady but stabilizes without assistance | 79355 (18.6%) | 69371 (20.3%) | 9984 (11.8%) | 55046 (22.4%) | 24309 (13.4%) |
| Not steady but stabilizes with assistance | 231248 (54.2%) | 187790 (54.9%) | 43458 (51.3%) | 139007 (56.6%) | 92241 (50.9%) |
| Did not walk | 101633 (23.8%) | 72063 (21.1%) | 29570 (34.9%) | 41620 (17.0%) | 60013 (33.1%) |
| Missing | 1361 (0.3%) | 1032 (0.3%) | 329 (0.4%) | 693 (0.3%) | 668 (0.4%) |
| Marital status |  |  |  |  |  |
| Married | 152827 (35.8%) | 121487 (35.5%) | 31340 (37.0%) | 91748 (37.4%) | 61079 (33.7%) |
| Not currently married | 257917 (60.4%) | 207648 (60.7%) | 50269 (59.3%) | 144622 (58.9%) | 113295 (62.5%) |
| Missing | 15936 (3.7%) | 12831 (3.8%) | 3105 (3.7%) | 9121 (3.7%) | 6815 (3.8%) |
| Level of understanding |  |  |  |  |  |
| Does not understand | 24397 (5.7%) | 14694 (4.3%) | 9703 (11.5%) | 7357 (3.0%) | 17040 (9.4%) |
| Understands | 401735 (94.2%) | 326948 (95.6%) | 74787 (88.3%) | 237954 (96.9%) | 163781 (90.4%) |
| Missing | 548 (0.1%) | 324 (0.1%) | 224 (0.3%) | 180 (0.1%) | 368 (0.2%) |
| Presence of delirium |  |  |  |  |  |
| No delirium | 403324 (94.5%) | 325399 (95.2%) | 77925 (92.0%) | 235353 (95.9%) | 167971 (92.7%) |
| Delirium | 9740 (2.3%) | 6146 (1.8%) | 3594 (4.2%) | 3142 (1.3%) | 6598 (3.6%) |
| Missing | 13616 (3.2%) | 10421 (3.0%) | 3195 (3.8%) | 6996 (2.8%) | 6620 (3.7%) |
| Depression |  |  |  |  |  |
| No depression | 405463 (95.0%) | 327074 (95.6%) | 78389 (92.5%) | 235808 (96.1%) | 169655 (93.6%) |
| Depression | 17724 (4.2%) | 12533 (3.7%) | 5191 (6.1%) | 8116 (3.3%) | 9608 (5.3%) |
| Missing | 3493 (0.8%) | 2359 (0.7%) | 1134 (1.3%) | 1567 (0.6%) | 1926 (1.1%) |
| Psychosis |  |  |  |  |  |
| No psychosis | 419955 (98.4%) | 337153 (98.6%) | 82802 (97.7%) | 242951 (99.0%) | 177004 (97.7%) |
| Psychosis | 5940 (1.4%) | 4311 (1.3%) | 1629 (1.9%) | 2240 (0.9%) | 3700 (2.0%) |
| Missing | 785 (0.2%) | 502 (0.1%) | 283 (0.3%) | 300 (0.1%) | 485 (0.3%) |
| Agitated and reactive behavior scale score |  |  |  |  |  |
| Score = 0 | 396880 (93.0%) | 321256 (93.9%) | 75624 (89.3%) | 233593 (95.2%) | 163287 (90.1%) |
| Score >0 | 28407 (6.7%) | 19726 (5.8%) | 8681 (10.2%) | 11219 (4.6%) | 17188 (9.5%) |
| Missing | 1393 (0.3%) | 984 (0.3%) | 409 (0.5%) | 679 (0.3%) | 714 (0.4%) |
| Bathing assistance |  |  |  |  |  |
| Needs assistance | 16220 (3.8%) | 14720 (4.3%) | 1500 (1.8%) | 12003 (4.9%) | 4217 (2.3%) |
| Independent | 409522 (96.0%) | 326503 (95.5%) | 83019 (98.0%) | 232954 (94.9%) | 176568 (97.4%) |
| Missing | 938 (0.2%) | 743 (0.2%) | 195 (0.2%) | 534 (0.2%) | 404 (0.2%) |
| Urinary catheter |  |  |  |  |  |
| No catheter | 387332 (90.8%) | 314648 (92.0%) | 72684 (85.8%) | 228598 (93.1%) | 158734 (87.6%) |
| Catheter present | 39339 (9.2%) | 27309 (8.0%) | 12030 (14.2%) | 16888 (6.9%) | 22451 (12.4%) |
| Missing | 9 (0.0%) | 9 (0.0%) | 0 (0%) | 5 (0.0%) | 4 (0.0%) |
| Ostomy |  |  |  |  |  |
| No ostomy | 418216 (98.0%) | 335586 (98.1%) | 82630 (97.5%) | 241185 (98.2%) | 177031 (97.7%) |
| Ostomy present | 8455 (2.0%) | 6371 (1.9%) | 2084 (2.5%) | 4301 (1.8%) | 4154 (2.3%) |
| Missing | 9 (0.0%) | 9 (0.0%) | 0 (0%) | 5 (0.0%) | 4 (0.0%) |
| Shortness of breath |  |  |  |  |  |
| Any shortness of breath | 74458 (17.5%) | 53170 (15.5%) | 21288 (25.1%) | 37681 (15.3%) | 36777 (20.3%) |
| No shortness of breath | 352106 (82.5%) | 288715 (84.4%) | 63391 (74.8%) | 207761 (84.6%) | 144345 (79.7%) |
| Missing | 116 (0.0%) | 81 (0.0%) | 35 (0.0%) | 49 (0.0%) | 67 (0.0%) |
| Fever |  |  |  |  |  |
| No fever | 420857 (98.6%) | 337528 (98.7%) | 83329 (98.4%) | 242502 (98.8%) | 178355 (98.4%) |
| Fever | 5807 (1.4%) | 4426 (1.3%) | 1381 (1.6%) | 2981 (1.2%) | 2826 (1.6%) |
| Missing | 16 (0.0%) | 12 (0.0%) | 4 (0.0%) | 8 (0.0%) | 8 (0.0%) |
| Vomiting |  |  |  |  |  |
| No vomiting | 420426 (98.5%) | 337415 (98.7%) | 83011 (98.0%) | 242411 (98.7%) | 178015 (98.2%) |
| Vomiting | 6242 (1.5%) | 4541 (1.3%) | 1701 (2.0%) | 3074 (1.3%) | 3168 (1.7%) |
| Missing | 12 (0.0%) | 10 (0.0%) | 2 (0.0%) | 6 (0.0%) | 6 (0.0%) |
| Dehydrated |  |  |  |  |  |
| No dehydration | 425597 (99.7%) | 341305 (99.8%) | 84292 (99.5%) | 245061 (99.8%) | 180536 (99.6%) |
| Dehydrated | 1063 (0.2%) | 647 (0.2%) | 416 (0.5%) | 422 (0.2%) | 641 (0.4%) |
| Missing | 20 (0.0%) | 14 (0.0%) | 6 (0.0%) | 8 (0.0%) | 12 (0.0%) |
| Internal bleeding |  |  |  |  |  |
| No bleeding | 424184 (99.4%) | 340300 (99.5%) | 83884 (99.0%) | 244405 (99.6%) | 179779 (99.2%) |
| Bleeding | 2482 (0.6%) | 1654 (0.5%) | 828 (1.0%) | 1078 (0.4%) | 1404 (0.8%) |
| Missing | 14 (0.0%) | 12 (0.0%) | 2 (0.0%) | 8 (0.0%) | 6 (0.0%) |
| Falls in past 6 months |  |  |  |  |  |
| No fall | 201136 (47.1%) | 160725 (47.0%) | 40411 (47.7%) | 118214 (48.2%) | 82922 (45.8%) |
| Fall | 205798 (48.2%) | 166804 (48.8%) | 38994 (46.0%) | 117860 (48.0%) | 87938 (48.5%) |
| Missing | 19746 (4.6%) | 14437 (4.2%) | 5309 (6.3%) | 9417 (3.8%) | 10329 (5.7%) |
| Trouble swallowing |  |  |  |  |  |
| Trouble swallowing | 21288 (5.0%) | 14087 (4.1%) | 7201 (8.5%) | 9240 (3.8%) | 12048 (6.6%) |
| No trouble swallowing | 404992 (94.9%) | 327624 (95.8%) | 77368 (91.3%) | 236095 (96.2%) | 168897 (93.2%) |
| Missing | 400 (0.1%) | 255 (0.1%) | 145 (0.2%) | 156 (0.1%) | 244 (0.1%) |
| Body mass index, kg/m^2^ |  |  |  |  |  |
| Mean (SD) | 27.2 (7.16) | 27.6 (7.20) | 25.5 (6.78) | 27.7 (7.13) | 26.6 (7.16) |
| Missing | 9339 (2.2%) | 6941 (2.0%) | 2398 (2.8%) | 4522 (1.8%) | 4817 (2.7%) |
| Weight loss |  |  |  |  |  |
| No weight loss | 408936 (95.8%) | 329051 (96.2%) | 79885 (94.3%) | 236181 (96.2%) | 172755 (95.3%) |
| Weight loss | 15892 (3.7%) | 11598 (3.4%) | 4294 (5.1%) | 8423 (3.4%) | 7469 (4.1%) |
| Missing | 1852 (0.4%) | 1317 (0.4%) | 535 (0.6%) | 887 (0.4%) | 965 (0.5%) |
| Artificial nutrition (e.g., parenteral/IV or tube feeding) |  |  |  |  |  |
| No | 413504 (96.9%) | 333531 (97.5%) | 79973 (94.4%) | 241123 (98.2%) | 172381 (95.1%) |
| Yes | 12746 (3.0%) | 8096 (2.4%) | 4650 (5.5%) | 4118 (1.7%) | 8628 (4.8%) |
| Missing | 430 (0.1%) | 339 (0.1%) | 91 (0.1%) | 250 (0.1%) | 180 (0.1%) |
| Pressure ulcer (stage 3, 4, or unstageable) |  |  |  |  |  |
| None | 402157 (94.3%) | 326574 (95.5%) | 75583 (89.2%) | 236597 (96.4%) | 165560 (91.4%) |
| Yes | 24523 (5.7%) | 15392 (4.5%) | 9131 (10.8%) | 8894 (3.6%) | 15629 (8.6%) |
| Tracheostomy care or invasive ventilator support within last 14 days |  |  |  |  |  |
| No | 420538 (98.6%) | 337300 (98.6%) | 83238 (98.3%) | 242454 (98.8%) | 178084 (98.3%) |
| Yes | 4963 (1.2%) | 3701 (1.1%) | 1262 (1.5%) | 2330 (0.9%) | 2633 (1.5%) |
| Missing | 1179 (0.3%) | 965 (0.3%) | 214 (0.3%) | 707 (0.3%) | 472 (0.3%) |
| Intravenous medication use |  |  |  |  |  |
| No | 399235 (93.6%) | 320488 (93.7%) | 78747 (93.0%) | 231116 (94.1%) | 168119 (92.8%) |
| Yes | 27308 (6.4%) | 21362 (6.2%) | 5946 (7.0%) | 14297 (5.8%) | 13011 (7.2%) |
| Missing | 137 (0.0%) | 116 (0.0%) | 21 (0.0%) | 78 (0.0%) | 59 (0.0%) |
| Dialysis |  |  |  |  |  |
| No | 415735 (97.4%) | 334517 (97.8%) | 81218 (95.9%) | 240945 (98.1%) | 174790 (96.5%) |
| Yes | 10822 (2.5%) | 7344 (2.1%) | 3478 (4.1%) | 4477 (1.8%) | 6345 (3.5%) |
| Missing | 123 (0.0%) | 105 (0.0%) | 18 (0.0%) | 69 (0.0%) | 54 (0.0%) |
| Chemotherapy or radiation therapy |  |  |  |  |  |
| No | 422534 (99.0%) | 339496 (99.3%) | 83038 (98.0%) | 243642 (99.2%) | 178892 (98.7%) |
| Yes | 4001 (0.9%) | 2349 (0.7%) | 1652 (2.0%) | 1769 (0.7%) | 2232 (1.2%) |
| Missing | 145 (0.0%) | 121 (0.0%) | 24 (0.0%) | 80 (0.0%) | 65 (0.0%) |
| Presence of pain |  |  |  |  |  |
| No | 187164 (43.9%) | 143663 (42.0%) | 43501 (51.4%) | 98853 (40.3%) | 88311 (48.7%) |
| Yes | 236003 (55.3%) | 195596 (57.2%) | 40407 (47.7%) | 144742 (59.0%) | 91261 (50.4%) |
| Missing | 3513 (0.8%) | 2707 (0.8%) | 806 (1.0%) | 1896 (0.8%) | 1617 (0.9%) |
| Anticoagulant use |  |  |  |  |  |
| No | 260271 (61.0%) | 206374 (60.3%) | 53897 (63.6%) | 146237 (59.6%) | 114034 (62.9%) |
| Yes | 166368 (39.0%) | 135563 (39.6%) | 30805 (36.4%) | 99237 (40.4%) | 67131 (37.1%) |
| Missing | 41 (0.0%) | 29 (0.0%) | 12 (0.0%) | 17 (0.0%) | 24 (0.0%) |
| Antipsychotic use |  |  |  |  |  |
| No | 393223 (92.2%) | 316472 (92.5%) | 76751 (90.6%) | 230737 (94.0%) | 162486 (89.7%) |
| Yes | 33417 (7.8%) | 25464 (7.4%) | 7953 (9.4%) | 14736 (6.0%) | 18681 (10.3%) |
| Missing | 40 (0.0%) | 30 (0.0%) | 10 (0.0%) | 18 (0.0%) | 22 (0.0%) |
| Hypnotic use |  |  |  |  |  |
| No | 409635 (96.0%) | 327914 (95.9%) | 81721 (96.5%) | 235019 (95.7%) | 174616 (96.4%) |
| Yes | 17003 (4.0%) | 14021 (4.1%) | 2982 (3.5%) | 10455 (4.3%) | 6548 (3.6%) |
| Missing | 42 (0.0%) | 31 (0.0%) | 11 (0.0%) | 17 (0.0%) | 25 (0.0%) |
| Diuretic use |  |  |  |  |  |
| No | 268790 (63.0%) | 219871 (64.3%) | 48919 (57.7%) | 156612 (63.8%) | 112178 (61.9%) |
| Yes | 157853 (37.0%) | 122067 (35.7%) | 35786 (42.2%) | 88865 (36.2%) | 68988 (38.1%) |
| Missing | 37 (0.0%) | 28 (0.0%) | 9 (0.0%) | 14 (0.0%) | 23 (0.0%) |

**Supplementary Table S6:** Univariable association between predictors and 6-month mortality

|  | **Alive at 6 months**  **(N, row percentage)** | **Dead at 6 months**  **(N, row percentage)** | **Univariable Odds Ratios** | |
| --- | --- | --- | --- | --- |
|  |  |  | OR | 95% CI |
| **Age (years, median (IQR))** | 81 (74, 87) | 84 (77, 90) | 1.04 | 1.04, 1.04 |
| **Sex** |  |  |  |  |
| Male | 120,307 (76%) | 38,984 (24%) | Ref | Ref |
| Female | 221,659 (83%) | 45,730 (17%) | 0.64 | 0.63, 0.65 |
| **Medicaid status** |  |  |  |  |
| No | 280,742 (80%) | 69,755 (20%) | Ref | Ref |
| Yes | 61,224 (80%) | 14,959 (20%) | 0.98 | 0.96, 1.00 |
| **Individual comorbidities** |  |  |  |  |
| Weight loss | 50,227 (67%) | 24,485 (33%) | 2.36 | 2.32, 2.40 |
| Heart failure | 112,975 (73%) | 41,462 (27%) | 1.94 | 1.91, 1.97 |
| Diabetes (complicated) | 102,828 (79%) | 28,016 (21%) | 1.15 | 1.13, 1.17 |
| Renal failure (severe) | 26,766 (68%) | 12,595 (32%) | 2.06 | 2.01, 2.10 |
| Liver failure (severe) | 3,391 (60%) | 2,253 (40%) | 2.73 | 2.58, 2.88 |
| Dementia | 89,184 (75%) | 29,634 (25%) | 1.52 | 1.50, 1.55 |
| Other neurologic disease | 69,221 (74%) | 23,750 (26%) | 1.54 | 1.51, 1.56 |
| Lung disease | 125,741 (76%) | 38,825 (24%) | 1.45 | 1.43, 1.48 |
| Paralysis | 32,342 (78%) | 8,985 (22%) | 1.14 | 1.11, 1.16 |
| Metastatic cancer | 8,315 (44%) | 10,613 (56%) | 5.75 | 5.58, 5.92 |
| Solid cancer | 39,361 (76%) | 12,771 (24%) | 1.36 | 1.34, 1.39 |
| Leukemia/lymphoma | 8,881 (67%) | 4,377 (33%) | 2.04 | 1.97, 2.12 |
| **Hospital length of stay (median (IQR))** | 6 (4, 9) | 7 (5, 11) | 1.04 | 1.04, 1.04 |
| **Admission type** |  |  |  |  |
| Elective | 63,967 (90%) | 6,988 (9.8%) | Ref | Ref |
| Urgent/emergent | 277,999 (78%) | 77,726 (22%) | 2.56 | 2.49, 2.63 |
| **Hospital discharge diagnosis** |  |  |  |  |
| Injury (e.g., fracture) | 86,521 (86%) | 13,829 (14%) | 0.51 | 0.49, 0.52 |
| Circulatory (e.g., heart failure, stroke) | 58,023 (76%) | 18,317 (24%) | Ref | Ref |
| Musculoskeletal (e.g., knee replacement) | 48,925 (93%) | 3,486 (6.7%) | 0.23 | 0.22, 0.23 |
| Neoplastic | 6,765 (53%) | 5,928 (47%) | 2.78 | 2.67, 2.89 |
| Other | 141,732 (77%) | 43,154 (23%) | 0.96 | 0.95, 0.98 |
| **Hospitalizations in the past year** |  |  |  |  |
| 0 | 219,772 (83%) | 45,209 (17%) | Ref | Ref |
| 1 | 73,320 (78%) | 20,567 (22%) | 1.36 | 1.34, 1.39 |
| 2 | 27,194 (74%) | 9,467 (26%) | 1.69 | 1.65, 1.74 |
| 3 | 11,379 (71%) | 4,659 (29%) | 1.99 | 1.92, 2.06 |
| ≥4 | 10,301 (68%) | 4,812 (32%) | 2.27 | 2.19, 2.35 |
| **ADL score (median (IQR), range 0-28)** | 18.0 (14.0, 19.0) | 19.0 (17.0, 20.0) | 1.14 | 1.14, 1.14 |
| **Cognitive status** |  |  |  |  |
| No impairment | 226,675 (85%) | 39,533 (15%) | — | — |
| Mild impairment | 68,660 (76%) | 21,243 (24%) | 1.77 | 1.74, 1.81 |
| Moderate impairment | 42,249 (68%) | 19,836 (32%) | 2.69 | 2.64, 2.75 |
| Severe impairment | 4,382 (52%) | 4,102 (48%) | 5.37 | 5.14, 5.61 |
| **Oxygen use** |  |  |  |  |
| Yes | 65,289 (69%) | 29,109 (31%) | 2.22 | 2.18, 2.26 |
| No | 276,677 (83%) | 55,605 (17%) |  |  |
| **Urinary incontinence** |  |  |  |  |
| Always continent | 129,643 (88%) | 17,904 (12%) | — | — |
| Occasionally incontinent | 100,899 (82%) | 21,638 (18%) | 1.55 | 1.52, 1.59 |
| Frequently incontinent | 71,389 (75%) | 24,187 (25%) | 2.45 | 2.40, 2.51 |
| Always incontinent | 18,892 (63%) | 11,039 (37%) | 4.23 | 4.11, 4.35 |
| Not rated (e.g., urinary catheter) | 21,143 (68%) | 9,946 (32%) | 3.41 | 3.31, 3.50 |
| **Bowel incontinence** |  |  |  |  |
| Always continent | 198,528 (86%) | 31,623 (14%) | — | — |
| Occasionally incontinent | 45,528 (80%) | 11,497 (20%) | 1.59 | 1.55, 1.62 |
| Frequently incontinent | 66,272 (74%) | 23,766 (26%) | 2.25 | 2.21, 2.29 |
| Always incontinent | 24,891 (61%) | 15,612 (39%) | 3.94 | 3.85, 4.03 |
| Not rated (e.g., ostomy) | 6,747 (75%) | 2,216 (25%) | 2.06 | 1.96, 2.17 |
| **Balance while walking** |  |  |  |  |
| Steady at all times | 11,715 (90%) | 1,373 (10%) | — | — |
| Not steady but stabilizes without assistance | 69,475 (87%) | 9,996 (13%) | 1.23 | 1.16, 1.30 |
| Not steady but stabilizes with assistance | 188,547 (81%) | 43,660 (19%) | 1.98 | 1.87, 2.09 |
| Did not walk | 72,229 (71%) | 29,685 (29%) | 3.51 | 3.31, 3.72 |
| **Marital status** |  |  |  |  |
| Married | 124,919 (79%) | 32,311 (21%) | — | — |
| Not currently married | 217,047 (81%) | 52,403 (19%) | 0.93 | 0.92, 0.95 |
| **Level of understanding** |  |  |  |  |
| Does not understand | 14,748 (60%) | 9,804 (40%) | — | — |
| Understands | 327,218 (81%) | 74,910 (19%) | 0.34 | 0.34, 0.35 |
| **Presence of delirium** |  |  |  |  |
| No delirium | 335,813 (81%) | 81,104 (19%) | — | — |
| Delirium | 6,153 (63%) | 3,610 (37%) | 2.43 | 2.33, 2.53 |
| **Depression** |  |  |  |  |
| No depression | 329,433 (81%) | 79,523 (19%) | — | — |
| Depression | 12,533 (71%) | 5,191 (29%) | 1.72 | 1.66, 1.77 |
| **Psychosis** |  |  |  |  |
| No psychosis | 337,655 (80%) | 83,085 (20%) | — | — |
| Psychosis | 4,311 (73%) | 1,629 (27%) | 1.54 | 1.45, 1.63 |
| **Agitated and reactive behavior scale score** |  |  |  |  |
| Score = 0 | 322,238 (81%) | 76,032 (19%) | — | — |
| Score >0 | 19,728 (69%) | 8,682 (31%) | 1.87 | 1.82, 1.92 |
| **Bathing assistance** |  |  |  |  |
| Needs assistance | 14,726 (91%) | 1,501 (9.3%) | — | — |
| Independent | 327,240 (80%) | 83,213 (20%) | 2.49 | 2.37, 2.63 |
| **Urinary catheter** |  |  |  |  |
| No catheter | 314,656 (81%) | 72,684 (19%) | — | — |
| Catheter present | 27,310 (69%) | 12,030 (31%) | 1.91 | 1.86, 1.95 |
| **Ostomy** |  |  |  |  |
| No ostomy | 335,595 (80%) | 82,630 (20%) | — | — |
| Ostomy present | 6,371 (75%) | 2,084 (25%) | 1.33 | 1.26, 1.40 |
| **Shortness of breath** |  |  |  |  |
| Any shortness of breath | 53,183 (71%) | 21,300 (29%) | — | — |
| No shortness of breath | 288,783 (82%) | 63,414 (18%) | 0.55 | 0.54, 0.56 |
| **Fever** |  |  |  |  |
| No fever | 337,540 (80%) | 83,333 (20%) | — | — |
| Fever | 4,426 (76%) | 1,381 (24%) | 1.26 | 1.19, 1.34 |
| **Vomiting** |  |  |  |  |
| No vomiting | 337,425 (80%) | 83,013 (20%) | — | — |
| Vomiting | 4,541 (73%) | 1,701 (27%) | 1.52 | 1.44, 1.61 |
| **Dehydrated** |  |  |  |  |
| No dehydration | 341,319 (80%) | 84,298 (20%) | — | — |
| Dehydrated | 647 (61%) | 416 (39%) | 2.6 | 2.30, 2.94 |
| **Internal bleeding** |  |  |  |  |
| No bleeding | 340,312 (80%) | 83,886 (20%) | — | — |
| Bleeding | 1,654 (67%) | 828 (33%) | 2.03 | 1.87, 2.21 |
| **Falls in past 6 months** |  |  |  |  |
| No fall | 170,252 (80%) | 43,789 (20%) | — | — |
| Fall | 171,714 (81%) | 40,925 (19%) | 0.93 | 0.91, 0.94 |
| **Trouble swallowing** |  |  |  |  |
| Trouble swallowing | 14,087 (66%) | 7,201 (34%) | — | — |
| No trouble swallowing | 327,879 (81%) | 77,513 (19%) | 0.46 | 0.45, 0.48 |
| **Body mass index, kg/m^2^** | 26 (23, 31) | 24 (21, 29) | 0.95 | 0.95, 0.95 |
| **Weight loss** |  |  |  |  |
| No weight loss | 330,368 (80%) | 80,420 (20%) | — | — |
| Weight loss | 11,598 (73%) | 4,294 (27%) | 1.52 | 1.47, 1.58 |
| **Artificial nutrition (e.g., parenteral/IV or tube feeding)** |  |  |  |  |
| No | 333,868 (81%) | 80,063 (19%) | — | — |
| Yes | 8,098 (64%) | 4,651 (36%) | 2.4 | 2.31, 2.48 |
| **Pressure ulcer (stage 3, 4, or unstageable)** |  |  |  |  |
| None | 326,574 (81%) | 75,583 (19%) | — | — |
| Yes | 15,392 (63%) | 9,131 (37%) | 2.56 | 2.49, 2.63 |
| **Tracheostomy care or invasive ventilator support within last 14 days** |  |  |  |  |
| No | 338,265 (80%) | 83,452 (20%) | — | — |
| Yes | 3,701 (75%) | 1,262 (25%) | 1.38 | 1.30, 1.47 |
| **Intravenous medication use** |  |  |  |  |
| No | 320,604 (80%) | 78,768 (20%) | — | — |
| Yes | 21,362 (78%) | 5,946 (22%) | 1.13 | 1.10, 1.17 |
| **Dialysis** |  |  |  |  |
| No | 334,621 (80%) | 81,236 (20%) | — | — |
| Yes | 7,345 (68%) | 3,478 (32%) | 1.95 | 1.87, 2.03 |
| **Chemotherapy or radiation therapy** |  |  |  |  |
| No | 339,617 (80%) | 83,062 (20%) | — | — |
| Yes | 2,349 (59%) | 1,652 (41%) | 2.88 | 2.70, 3.06 |
| **Presence of pain** |  |  |  |  |
| No | 144,775 (77%) | 43,935 (23%) | — | — |
| Yes | 197,191 (83%) | 40,779 (17%) | 0.68 | 0.67, 0.69 |
| **Anticoagulant use** |  |  |  |  |
| No | 206,397 (79%) | 53,907 (21%) | — | — |
| Yes | 135,569 (81%) | 30,807 (19%) | 0.87 | 0.86, 0.88 |
| **Antipsychotic use** |  |  |  |  |
| No | 316,501 (80%) | 76,761 (20%) | — | — |
| Yes | 25,465 (76%) | 7,953 (24%) | 1.29 | 1.25, 1.32 |
| **Hypnotic use** |  |  |  |  |
| No | 327,945 (80%) | 81,732 (20%) | — | — |
| Yes | 14,021 (82%) | 2,982 (18%) | 0.85 | 0.82, 0.89 |
| **Diuretic use** |  |  |  |  |
| No | 219,897 (82%) | 48,922 (18%) | — | — |
| Yes | 122,069 (77%) | 35,792 (23%) | 1.32 | 1.30, 1.34 |

Abbreviations: ADL, activities of daily living; CI, confidence interval; IQR, interquartile range; OR, odds ratio; Ref, reference

**Supplementary Table S7**: Univariable associations between predictors and successful community discharge

|  | **Did not experience a successful community discharge**  **(N, row percentage)** | **Successful community discharge**  **(N, row percentage)** | **Univariable Odds Ratios** | |
| --- | --- | --- | --- | --- |
|  |  |  | OR | 95% CI |
| **Age (years, median (IQR))** | 82 (75, 89) | 81 (74, 87) | 0.98 | 0.98, 0.98 |
| **Sex** |  |  |  |  |
| Male | 73,235 (46%) | 86,056 (54%) | — | — |
| Female | 107,954 (40%) | 159,435 (60%) | 1.26 | 1.24, 1.27 |
| **Medicaid status** |  |  |  |  |
| No | 139,524 (40%) | 210,973 (60%) | — | — |
| Yes | 41,665 (55%) | 34,518 (45%) | 0.55 | 0.54, 0.56 |
| **Individual comorbidities** |  |  |  |  |
| Weight loss | 41,180 (55%) | 33,532 (45%) | 0.54 | 0.53, 0.55 |
| Heart failure | 76,206 (49%) | 78,231 (51%) | 0.64 | 0.64, 0.65 |
| Diabetes (complicated) | 60,138 (46%) | 70,706 (54%) | 0.81 | 0.80, 0.83 |
| Renal failure (severe) | 21,564 (55%) | 17,797 (45%) | 0.58 | 0.57, 0.59 |
| Liver failure (severe) | 3,226 (57%) | 2,418 (43%) | 0.55 | 0.52, 0.58 |
| Dementia | 64,360 (54%) | 54,458 (46%) | 0.52 | 0.51, 0.52 |
| Other neurologic disease | 48,987 (53%) | 43,984 (47%) | 0.59 | 0.58, 0.60 |
| Lung disease | 74,940 (46%) | 89,626 (54%) | 0.82 | 0.81, 0.83 |
| Paralysis | 21,187 (51%) | 20,140 (49%) | 0.67 | 0.66, 0.69 |
| Metastatic cancer | 11,632 (61%) | 7,296 (39%) | 0.45 | 0.43, 0.46 |
| Solid cancer | 22,916 (44%) | 29,216 (56%) | 0.93 | 0.92, 0.95 |
| Leukemia/lymphoma | 6,648 (50%) | 6,610 (50%) | 0.73 | 0.70, 0.75 |
| **Hospital length of stay (median (IQR))** | 7 (5, 11) | 6 (4, 8) | 0.95 | 0.95, 0.95 |
| **Admission type** |  |  |  |  |
| Elective | 20,304 (29%) | 50,651 (71%) | — | — |
| Urgent/emergent | 160,885 (45%) | 194,840 (55%) | 0.49 | 0.48, 0.49 |
| **Hospital discharge diagnosis** |  |  |  |  |
| Injury (e.g., fracture) | 37,585 (37%) | 62,765 (63%) | 1.49 | 1.46, 1.52 |
| Circulatory (e.g., heart failure, stroke) | 36,001 (47%) | 40,339 (53%) | Ref | Ref |
| Musculoskeletal (e.g., knee replacement) | 11,663 (22%) | 40,748 (78%) | 3.12 | 3.04, 3.20 |
| Neoplastic | 7,162 (56%) | 5,531 (44%) | 0.69 | 0.66, 0.72 |
| Other | 88,778 (48%) | 96,108 (52%) | 0.97 | 0.95, 0.98 |
| **Hospitalizations in the past year** |  |  |  |  |
| 0 | 102,473 (39%) | 162,508 (61%) | — | — |
| 1 | 42,040 (45%) | 51,847 (55%) | 0.78 | 0.77, 0.79 |
| 2 | 18,547 (51%) | 18,114 (49%) | 0.62 | 0.60, 0.63 |
| 3 | 8,744 (55%) | 7,294 (45%) | 0.53 | 0.51, 0.54 |
| ≥4 | 9,385 (62%) | 5,728 (38%) | 0.38 | 0.37, 0.40 |
| **ADL score (median (IQR), range 0-28)** | 19.0 (17.0-20.0) | 17.0 (14.0-19.0) | 0.89 | 0.89, 0.89 |
| **Cognitive status** |  |  |  |  |
| No impairment | 91,905 (35%) | 174,303 (65%) | — | — |
| Mild impairment | 44,235 (49%) | 45,668 (51%) | 0.54 | 0.54, 0.55 |
| Moderate impairment | 38,483 (62%) | 23,602 (38%) | 0.32 | 0.32, 0.33 |
| Severe impairment | 6,566 (77%) | 1,918 (23%) | 0.15 | 0.15, 0.16 |
| **Oxygen use** |  |  |  |  |
| Yes | 48,488 (51%) | 45,910 (49%) | 0.63 | 0.62, 0.64 |
| No | 132,701 (40%) | 199,581 (60%) | — | — |
| **Urinary incontinence** |  |  |  |  |
| Always continent | 43,246 (29%) | 104,301 (71%) | — | — |
| Occasionally incontinent | 48,055 (39%) | 74,482 (61%) | 0.64 | 0.63, 0.65 |
| Frequently incontinent | 50,959 (53%) | 44,617 (47%) | 0.36 | 0.36, 0.37 |
| Always incontinent | 20,611 (69%) | 9,320 (31%) | 0.19 | 0.18, 0.19 |
| Not rated (e.g., urinary catheter) | 18,318 (59%) | 12,771 (41%) | 0.29 | 0.28, 0.30 |
| **Bowel incontinence** |  |  |  |  |
| Always continent | 75,407 (33%) | 154,744 (67%) | — | — |
| Occasionally incontinent | 24,886 (44%) | 32,139 (56%) | 0.63 | 0.62, 0.64 |
| Frequently incontinent | 48,223 (54%) | 41,815 (46%) | 0.42 | 0.42, 0.43 |
| Always incontinent | 28,281 (70%) | 12,222 (30%) | 0.21 | 0.21, 0.22 |
| Not rated (e.g., ostomy) | 4,392 (49%) | 4,571 (51%) | 0.51 | 0.49, 0.53 |
| **Balance while walking** |  |  |  |  |
| Steady at all times | 3,959 (30%) | 9,129 (70%) | — | — |
| Not steady but stabilizes without assistance | 24,345 (31%) | 55,126 (69%) | 0.98 | 0.94, 1.02 |
| Not steady but stabilizes with assistance | 92,674 (40%) | 139,533 (60%) | 0.65 | 0.63, 0.68 |
| Did not walk | 60,211 (59%) | 41,703 (41%) | 0.3 | 0.29, 0.31 |
| **Marital status** |  |  |  |  |
| Married | 62,842 (40%) | 94,388 (60%) | — | — |
| Not currently married | 118,347 (44%) | 151,103 (56%) | 0.85 | 0.84, 0.86 |
| **Level of understanding** |  |  |  |  |
| Does not understand | 17,185 (70%) | 7,367 (30%) | — | — |
| Understands | 164,004 (41%) | 238,124 (59%) | 3.39 | 3.29, 3.48 |
| **Presence of delirium** |  |  |  |  |
| No delirium | 174,569 (42%) | 242,348 (58%) | — | — |
| Delirium | 6,620 (68%) | 3,143 (32%) | 0.34 | 0.33, 0.36 |
| **Depression** |  |  |  |  |
| No depression | 171,581 (42%) | 237,375 (58%) | — | — |
| Depression | 9,608 (54%) | 8,116 (46%) | 0.61 | 0.59, 0.63 |
| **Psychosis** |  |  |  |  |
| No psychosis | 177,489 (42%) | 243,251 (58%) | — | — |
| Psychosis | 3,700 (62%) | 2,240 (38%) | 0.44 | 0.42, 0.47 |
| **Agitated and reactive behavior scale score** |  |  |  |  |
| Score = 0 | 163,999 (41%) | 234,271 (59%) | — | — |
| Score >0 | 17,190 (61%) | 11,220 (39%) | 0.46 | 0.45, 0.47 |
| **Bathing assistance** |  |  |  |  |
| Needs assistance | 4,220 (26%) | 12,007 (74%) | — | — |
| Independent | 176,969 (43%) | 233,484 (57%) | 0.46 | 0.45, 0.48 |
| **Urinary catheter** |  |  |  |  |
| No catheter | 158,738 (41%) | 228,602 (59%) | — | — |
| Catheter present | 22,451 (57%) | 16,889 (43%) | 0.52 | 0.51, 0.53 |
| **Ostomy** |  |  |  |  |
| No ostomy | 177,035 (42%) | 241,190 (58%) | — | — |
| Ostomy present | 4,154 (49%) | 4,301 (51%) | 0.76 | 0.73, 0.79 |
| **Shortness of breath** |  |  |  |  |
| Any shortness of breath | 36,795 (49%) | 37,688 (51%) | — | — |
| No shortness of breath | 144,394 (41%) | 207,803 (59%) | 1.41 | 1.38, 1.43 |
| **Fever** |  |  |  |  |
| No fever | 178,363 (42%) | 242,510 (58%) | — | — |
| Fever | 2,826 (49%) | 2,981 (51%) | 0.78 | 0.74, 0.82 |
| **Vomiting** |  |  |  |  |
| No vomiting | 178,021 (42%) | 242,417 (58%) | — | — |
| Vomiting | 3,168 (51%) | 3,074 (49%) | 0.71 | 0.68, 0.75 |
| **Dehydrated** |  |  |  |  |
| No dehydration | 180,548 (42%) | 245,069 (58%) | — | — |
| Dehydrated | 641 (60%) | 422 (40%) | 0.49 | 0.43, 0.55 |
| **Internal bleeding** |  |  |  |  |
| No bleeding | 179,785 (42%) | 244,413 (58%) | — | — |
| Bleeding | 1,404 (57%) | 1,078 (43%) | 0.56 | 0.52, 0.61 |
| **Falls in past 6 months** |  |  |  |  |
| No fall | 89,382 (42%) | 124,659 (58%) | — | — |
| Fall | 91,807 (43%) | 120,832 (57%) | 0.94 | 0.93, 0.96 |
| **Trouble swallowing** |  |  |  |  |
| Trouble swallowing | 12,048 (57%) | 9,240 (43%) | — | — |
| No trouble swallowing | 169,141 (42%) | 236,251 (58%) | 1.82 | 1.77, 1.87 |
| **Body mass index, kg/m^2^** | 25 (22, 30) | 26 (23, 31) | 1.02 | 1.02, 1.02 |
| **Weight loss** |  |  |  |  |
| No weight loss | 173,720 (42%) | 237,068 (58%) | — | — |
| Weight loss | 7,469 (47%) | 8,423 (53%) | 0.83 | 0.80, 0.85 |
| **Artificial nutrition (e.g., parenteral/IV or tube feeding)** |  |  |  |  |
| No | 172,559 (42%) | 241,372 (58%) | — | — |
| Yes | 8,630 (68%) | 4,119 (32%) | 0.34 | 0.33, 0.35 |
| **Pressure ulcer (stage 3, 4, or unstageable)** |  |  |  |  |
| None | 165,560 (41%) | 236,597 (59%) | — | — |
| Yes | 15,629 (64%) | 8,894 (36%) | 0.4 | 0.39, 0.41 |
| **Tracheostomy care or invasive ventilator support within last 14 days** |  |  |  |  |
| No | 178,556 (42%) | 243,161 (58%) | — | — |
| Yes | 2,633 (53%) | 2,330 (47%) | 0.65 | 0.61, 0.69 |
| **Intravenous medication use** |  |  |  |  |
| No | 168,178 (42%) | 231,194 (58%) | — | — |
| Yes | 13,011 (48%) | 14,297 (52%) | 0.8 | 0.78, 0.82 |
| **Dialysis** |  |  |  |  |
| No | 174,843 (42%) | 241,014 (58%) | — | — |
| Yes | 6,346 (59%) | 4,477 (41%) | 0.51 | 0.49, 0.53 |
| **Chemotherapy or radiation therapy** |  |  |  |  |
| No | 178,957 (42%) | 243,722 (58%) | — | — |
| Yes | 2,232 (56%) | 1,769 (44%) | 0.58 | 0.55, 0.62 |
| **Presence of pain** |  |  |  |  |
| No | 89,139 (47%) | 99,571 (53%) | — | — |
| Yes | 92,050 (39%) | 145,920 (61%) | 1.42 | 1.40, 1.44 |
| **Anticoagulant use** |  |  |  |  |
| No | 114,054 (44%) | 146,250 (56%) | — | — |
| Yes | 67,135 (40%) | 99,241 (60%) | 1.15 | 1.14, 1.17 |
| **Antipsychotic use** |  |  |  |  |
| No | 162,507 (41%) | 230,755 (59%) | — | — |
| Yes | 18,682 (56%) | 14,736 (44%) | 0.56 | 0.54, 0.57 |
| **Hypnotic use** |  |  |  |  |
| No | 174,641 (43%) | 235,036 (57%) | — | — |
| Yes | 6,548 (39%) | 10,455 (61%) | 1.19 | 1.15, 1.22 |
| **Diuretic use** |  |  |  |  |
| No | 112,195 (42%) | 156,624 (58%) | — | — |
| Yes | 68,994 (44%) | 88,867 (56%) | 0.92 | 0.91, 0.93 |

Abbreviations: ADL, activities of daily living; CI, confidence interval; IQR, interquartile range; OR, odds ratio; Ref, reference

**Supplementary Table S8:** Full model coefficients for the final updated Minimum Data Set model for predicting 6-month mortality*

|  | **Βeta-coefficient** | **S.E.** | **Wald *Z*** | **P-value** |
| --- | --- | --- | --- | --- |
| **Intercept** | -5.963 | 0.191 | -31.191 | 0.000 |
| **age** | 0.021 | 0.002 | 8.381 | 0.000 |
| **age'** | 0.019 | 0.006 | 2.911 | 0.004 |
| **age''** | -0.007 | 0.023 | -0.326 | 0.745 |
| **Male sex (vs. female)** | 0.338 | 0.009 | 37.838 | 0.000 |
| **Medicaid enrollment (vs. not)** | -0.218 | 0.012 | -18.885 | 0.000 |
| **Comorbidities** |  |  |  |  |
| Weight loss | 0.481 | 0.010 | 46.184 | 0.000 |
| Heart failure | 0.370 | 0.010 | 38.105 | 0.000 |
| Diabetes (complicated) | 0.032 | 0.010 | 3.268 | 0.001 |
| Renal failure (severe) | 0.524 | 0.013 | 38.986 | 0.000 |
| Liver failure (severe) | 1.091 | 0.031 | 34.878 | 0.000 |
| Dementia | -0.031 | 0.011 | -2.824 | 0.005 |
| Other neurologic disease | -0.027 | 0.010 | -2.585 | 0.010 |
| Lung disease | 0.198 | 0.010 | 20.737 | 0.000 |
| Paralysis | -0.245 | 0.015 | -16.297 | 0.000 |
| Metastatic cancer | 1.791 | 0.019 | 96.489 | 0.000 |
| Solid cancer | 0.326 | 0.013 | 25.747 | 0.000 |
| Leukemia/lymphoma | 0.514 | 0.022 | 23.802 | 0.000 |
| **Hospital length of stay** | 0.040 | 0.009 | 4.589 | 0.000 |
| **Hospital length of stay'** | -0.310 | 0.277 | -1.118 | 0.264 |
| **Hospital length of stay''** | 0.334 | 0.377 | 0.886 | 0.376 |
| **Hospital discharge diagnoses (reference = Other)** |  |  |  |  |
| Injury (e.g., fractures) | -0.475 | 0.012 | -39.768 | 0.000 |
| Circulatory | 0.130 | 0.012 | 11.296 | 0.000 |
| Musculoskeletal system | -0.529 | 0.021 | -25.588 | 0.000 |
| Neoplasms | 0.764 | 0.024 | 32.122 | 0.000 |
| **Admission type (urgent/emergent vs. elective)** | 0.535 | 0.016 | 33.757 | 0.000 |
| **Hospitalizations in the past year (reference = 0 hospitalizations)** |  |  |  |  |
| 1 | 0.111 | 0.011 | 10.315 | 0.000 |
| 2 | 0.207 | 0.015 | 13.748 | 0.000 |
| 3 | 0.303 | 0.021 | 14.474 | 0.000 |
| 4+ | 0.325 | 0.022 | 14.986 | 0.000 |
| **MDS-ADL score** | 0.018 | 0.004 | 4.846 | 0.000 |
| **MDS-ADL score’** | 0.039 | 0.006 | 7.038 | 0.000 |
| **MDS-ADL score’’** | -0.149 | 0.066 | -2.265 | 0.024 |
| **Cognitive function scale (reference = no impairment)** |  |  |  |  |
| Mild impairment | 0.333 | 0.011 | 30.070 | 0.000 |
| Moderate impairment | 0.676 | 0.014 | 49.543 | 0.000 |
| Severe impairment | 1.072 | 0.027 | 39.064 | 0.000 |
| **Oxygen use** | 0.548 | 0.010 | 54.642 | 0.000 |
| **Urinary incontinence (reference = always continent)** |  |  |  |  |
| Occasionally incontinent | 0.104 | 0.013 | 8.039 | 0.000 |
| Frequently incontinent | 0.167 | 0.016 | 10.688 | 0.000 |
| Always incontinent | 0.184 | 0.023 | 8.067 | 0.000 |
| Not rated (e.g., urinary catheter) | 0.334 | 0.018 | 18.075 | 0.000 |
| **Bowel incontinence (reference = always continent)** |  |  |  |  |
| Occasionally incontinent | 0.141 | 0.014 | 9.968 | 0.000 |
| Frequently incontinent | 0.253 | 0.013 | 18.940 | 0.000 |
| Always incontinent | 0.444 | 0.019 | 22.856 | 0.000 |
| Not rated (e.g., ostomy) | 0.118 | 0.029 | 4.085 | 0.000 |
| **Balance while walking (reference = steady at all times)** |  |  |  |  |
| Not steady but stabilizes without assistance | 0.054 | 0.033 | 1.621 | 0.105 |
| Not steady but stabilizes with assistance | 0.179 | 0.033 | 5.435 | 0.000 |
| Did not walk | 0.419 | 0.034 | 12.270 | 0.000 |

Abbreviations: MDS-ADL, Minimum Data Set-Activities of Daily Living; S.E., standard error

* Age was modeled continuously as a restricted cubic spline with 4 knots placed at ages 68, 78, 85, and 94 years. Hospital length of stay was modeled continuously as a restricted cubic spline with 4 knots placed at 4, 5, 8, and 21 days. ADL score was modeled continuously as a restricted cubic spline with 4 knots placed at 9, 17, 19, and 22.

**Supplementary Table S9:** Full model coefficients for the final updated Minimum Data Set model for predicting successful community discharge*

|  | **Βeta-coefficient** | **S.E.** | **Wald *Z*** | **P-value** |
| --- | --- | --- | --- | --- |
| **Intercept** | 2.020 | 0.139 | 14.551 | 0.000 |
| **age** | 0.002 | 0.002 | 1.185 | 0.236 |
| **age'** | -0.021 | 0.005 | -4.355 | 0.000 |
| **age''** | 0.023 | 0.018 | 1.272 | 0.203 |
| **Male sex (vs. female)** | -0.107 | 0.007 | -14.863 | 0.000 |
| **Medicaid enrollment (vs. not)** | -0.434 | 0.009 | -48.298 | 0.000 |
| **Comorbidities** |  |  |  |  |
| Weight loss | -0.215 | 0.009 | -23.595 | 0.000 |
| Heart failure | -0.169 | 0.008 | -21.498 | 0.000 |
| Diabetes (complicated) | -0.050 | 0.008 | -6.529 | 0.000 |
| Renal failure (severe) | -0.270 | 0.012 | -22.666 | 0.000 |
| Liver failure (severe) | -0.469 | 0.029 | -15.982 | 0.000 |
| Dementia | -0.182 | 0.009 | -20.625 | 0.000 |
| Other neurologic disease | -0.003 | 0.009 | -0.310 | 0.757 |
| Lung disease | -0.075 | 0.008 | -9.819 | 0.000 |
| Paralysis | 0.110 | 0.012 | 9.032 | 0.000 |
| Metastatic cancer | -0.707 | 0.018 | -39.835 | 0.000 |
| Solid cancer | -0.069 | 0.011 | -6.478 | 0.000 |
| Leukemia/lymphoma | -0.223 | 0.019 | -11.597 | 0.000 |
| **Hospital length of stay** | -0.042 | 0.007 | -6.307 | 0.000 |
| **Hospital length of stay'** | 0.089 | 0.216 | 0.411 | 0.681 |
| **Hospital length of stay''** | -0.074 | 0.295 | -0.252 | 0.801 |
| **Admission type (urgent/emergent vs. elective)** | -0.309 | 0.011 | -27.778 | 0.000 |
| **Hospital discharge diagnoses (reference = other)** |  |  |  |  |
| Injury (e.g., fractures) | 0.288 | 0.009 | 31.905 | 0.000 |
| Circulatory (e.g., heart failure, stroke) | -0.052 | 0.010 | -5.426 | 0.000 |
| Musculoskeletal system | 0.374 | 0.014 | 27.271 | 0.000 |
| Neoplasms | -0.330 | 0.022 | -15.029 | 0.000 |
| **Hospitalizations in the past year (reference = 0 hospitalizations)** |  |  |  |  |
| 1 | -0.104 | 0.009 | -12.186 | 0.000 |
| 2 | -0.265 | 0.012 | -21.193 | 0.000 |
| 3 | -0.354 | 0.018 | -19.576 | 0.000 |
| 4+ | -0.584 | 0.019 | -30.193 | 0.000 |
| **MDS-ADL score** | -0.001 | 0.003 | -0.588 | 0.556 |
| **MDS-ADL score’** | -0.044 | 0.004 | -11.142 | 0.000 |
| **MDS-ADL score’’** | 0.190 | 0.053 | 3.559 | 0.000 |
| **Cognitive function scale (reference = no impairment)** |  |  |  |  |
| Mild impairment | -0.304 | 0.009 | -34.599 | 0.000 |
| Moderate impairment | -0.577 | 0.012 | -49.574 | 0.000 |
| Severe impairment | -0.847 | 0.029 | -28.984 | 0.000 |
| **Oxygen use** | -0.241 | 0.009 | -27.943 | 0.000 |
| **Urinary incontinence (reference = always continent)** |  |  |  |  |
| Occasionally incontinent | -0.158 | 0.009 | -16.707 | 0.000 |
| Frequently incontinent | -0.304 | 0.012 | -25.325 | 0.000 |
| Always incontinent | -0.333 | 0.020 | -16.472 | 0.000 |
| Not rated (e.g., urinary catheter) | -0.476 | 0.015 | -31.310 | 0.000 |
| **Bowel incontinence (reference = always continent)** |  |  |  |  |
| Occasionally incontinent | -0.111 | 0.011 | -10.273 | 0.000 |
| Frequently incontinent | -0.190 | 0.011 | -17.899 | 0.000 |
| Always incontinent | -0.371 | 0.017 | -21.279 | 0.000 |
| Not rated (e.g., ostomy) | -0.123 | 0.024 | -5.185 | 0.000 |
| **Balance while walking (reference = steady at all times)** |  |  |  |  |
| Not steady but stabilizes without assistance | 0.069 | 0.022 | 3.138 | 0.002 |
| Not steady but stabilizes with assistance | -0.033 | 0.022 | -1.496 | 0.135 |
| Did not walk | -0.411 | 0.023 | -17.609 | 0.000 |

Abbreviations: MDS-ADL, Minimum Data Set-Activities of Daily Living; S.E., standard error

* Age was modeled continuously as a restricted cubic spline with 4 knots placed at ages 68, 78, 85, and 94 years. Hospital length of stay was modeled continuously as a restricted cubic spline with 4 knots placed at 4, 5, 8, and 21 days. ADL score was modeled continuously as a restricted cubic spline with 4 knots placed at 9, 17, 19, and 22.

**Supplementary Table S10:** Apparent and optimism-corrected performance following bootstrap internal validation of the base model and updated Minimum Data Set model for predicting 6-month mortality and successful community discharge

|  | **Day 7 base model without MDS** | | **Day 7 MDS model** | |
| --- | --- | --- | --- | --- |
|  | Apparent performance (95% CI) | Optimism-corrected performance (95% CI) | Apparent performance (95% CI) | Optimism-corrected performance (95% CI) |
| **6-month mortality** |  |  |  |  |
| c-statistic | 0.747  (0.745, 0.749) | 0.747  (0.745, 0.749) | 0.789  (0.787, 0.790) | 0.789  (0.787, 0.790) |
| Calibration in the large (intercept) | 0.000  (0.000, 0.000) | -0.001  (-0.003, 0.001) | 0.000  (0.000, 0.000) | -0.001  (-0.002, 0.000) |
| Calibration slope | 1.000  (1.000, 1.000) | 0.999  (0.998, 1.001) | 1.000 (1.000, 1.000) | 0.999  (0.998, 1.000) |
| Integrated calibration index (Eavg) | 0.012  (0.011, 0.012) | 0.012  (0.011, 0.012) | 0.011 (0.010, 0.012) | 0.011  (0.010, 0.012) |
| Brier score | 0.139  (0.138, 0.140) | 0.139  (0.138, 0.140) | 0.130 (0.130, 0.131) | 0.130  (0.130, 0.131) |
| **Successful community discharge** |  |  |  |  |
| c-statistic | 0.685  (0.684, 0.686) | 0.685  (0.683, 0.687) | 0.730  (0.728, 0.731) | 0.730  (0.728, 0.731) |
| Calibration in the large (intercept) | 0.000  (0.000, 0.000) | 0.000  (-0.001, 0.001) | 0.000  (0.000, 0.000) | 0.000  (0.000, 0.001) |
| Calibration slope | 1.000  (1.000, 1.000) | 0.999  (0.998, 1.000) | 1.000  (1.000, 1.000) | 0.999  (0.998, 1.000) |
| Integrated calibration index (Eavg) | 0.013  (0.012, 0.014) | 0.013  (0.012, 0.014) | 0.011  (0.010, 0.012) | 0.011  (0.010, 0.012) |
| Brier score | 0.219  (0.219, 0.220) | 0.219  (0.219, 0.220) | 0.206  (0.206, 0.207) | 0.206  (0.206, 0.207) |

Abbreviations: c-statistic, concordance statistic; CI, confidence interval; Eavg, the average absolute difference between observed and predicted probabilities over the range of predicted probabilities; MDS, Minimum Data Set

**Supplementary Table S11**: Additional performance measures to assess the value of adding Minimum Data Set data elements to the base model

|  |  | **Value** | **Formula** |
| --- | --- | --- | --- |
| **6-month mortality** | |  |  |
| A | Likelihood ratio X^2^ of Base Model | 54839.41 |  |
| B | Likelihood ratio X^2^ of MDS Model | 76387.20 |  |
| C | Adequacy of Base Model | 0.72 | A / B |
| D | Fraction of new information from MDS data | 0.28 | 1 – C |
| E | Pseudo R^2^ of Base Model | 0.191 |  |
| F | Pseudo R^2^ of MDS Model | 0.260 |  |
| G | Variance of Base model risk | 0.022 |  |
| H | Variance of MDS model risk | 0.030 |  |
| I | Relative explained variation | 0.71 | G / H |
| J | Fraction of new information | 0.29 | 1 – I |
| K | Base Model fraction explained risk | 0.136 |  |
| L | MDS model fraction explained risk | 0.191 |  |
| M | Relative explained variation | 0.71 | K / L |
| N | Fraction of new information | 0.29 | 1 – M |
| **Successful community discharge** | |  |  |
| A | Likelihood ratio X^2^ of Base Model | 46,398.84 |  |
| B | Likelihood ratio X^2^ of MDS Model | 71,602.09 |  |
| C | Adequacy of Base Model | 0.65 | A / B |
| D | Fraction of new information from MDS data | 0.35 | 1 – C |
| E | Pseudo R^2^ of Base Model | 0.138 |  |
| F | Pseudo R2 of MDS Model | 0.208 |  |
| G | Variance of Base model risk | 0.025 |  |
| H | Variance of MDS model risk | 0.038 |  |
| I | Relative explained variation | 0.66 | G / H |
| J | Fraction of new information | 0.37 | 1 – I |
| K | Base Model fraction explained risk | 0.104 |  |
| L | MDS model fraction explained risk | 0.158 |  |
| M | Relative explained variation | 0.66 | K / L |
| N | Fraction of new information | 0.37 | 1 - M |

Note: The adequacy of the base model is calculated as the likelihood ratio X^2^ of the base model divided by the likelihood ratio X^2^ of the MDS model (i.e., in this table taking the value of column A divided by column B). It refers to the proportion of log likelihood explained by the base model with reference to the log likelihood explained by the entire set (base model + MDS predictors). A value of 1 would indicate that the base model contains all the predictive information found in the whole set of predictors (base + MDS predictors). The fraction of new information refers to 1 minus the adequacy index which indicates the proportion of explainable variation that is explained by MDS predictors. Nagelkerke’s pseudo R^2^ assesses the goodness of fit for logistic regression models with a value of 1 indicating perfect model fit. Relative explained variation is the ratio of the variances of predicted values from the base model to the MDS model. The fraction of new information refers to 1 minus this number. The fraction explained risk is the R^2^ measure for a binary outcome, calculated as variance of predicted risk divided by the sum of the variance of predicted risk and the average risk times (1 – risk). Relative explained variation is the ratio of the fraction explained risk of the base model to the MDS model. The fraction of new information is 1 minus this number. The goal of these values is to capture the proportion of predictive information in the model that was added by MDS predictors.

**Supplementary Table S12:** Optimism-corrected performance of the model for the outcome of 6-month mortality across subgroups by age, sex, race and ethnicity, and clinical diagnosis

| **Outcome: 6-month mortality** | | | | |
| --- | --- | --- | --- | --- |
| **Subgroup** | **c-statistic (95% CI)** | **Calibration intercept (95% CI)** | **Calibration slope (95% CI)** | **ICI (Eavg) (95% CI)** |
| **Age group** |  |  |  |  |
| 66-74 | 0.824 (0.821, 0.828) | 0.099 (0.074, 0.123) | 1.071 (1.056, 1.087) | 0.010 (0.008, 0.011) |
| 75-84 | 0.796 (0.793, 0.799) | 0.029 (0.012, 0.046) | 1.023 (1.012, 1.034) | 0.012 (0.010, 0.013) |
| 85+ | 0.746 (0.743, 0.749) | -0.063 (-0.073, -0.054) | 0.934 (0.924, 0.943) | 0.012 (0.011, 0.014) |
| **Sex** |  |  |  |  |
| Male | 0.774 (0.772, 0.777) | -0.033 (-0.042, -0.023) | 0.965 (0.955, 0.974) | 0.013 (0.011, 0.014) |
| Female | 0.791 (0.789, 0.793) | 0.030 (0.021, 0.040) | 1.024 (1.016, 1.031) | 0.010 (0.010, 0.011) |
| **Race and ethnicity^a^** |  |  |  |  |
| Asian/Pacific Islander | 0.793 (0.782, 0.805) | -0.388 (-0.473, -0.304) | 0.943 (0.887, 0.999) | 0.044 (0.036, 0.052) |
| Black | 0.792 (0.786, 0.797) | -0.234 (-0.269, -0.199) | 0.975 (0.951, 0.999) | 0.029 (0.025, 0.033) |
| Hispanic | 0.783 (0.774, 0.792) | -0.301 (-0.364, -0.238) | 0.938 (0.896, 0.980) | 0.031 (0.026, 0.037) |
| American Indian/Alaska Native | 0.790 (0.765, 0.814) | 0.027 (-0.176, 0.231) | 1.035 (0.900, 1.169) | 0.005 (-0.004, 0.013) |
| Other | 0.781 (0.754, 0.808) | -0.433 (-0.592, -0.273) | 0.883 (0.769, 0.997) | 0.036 (0.024, 0.049) |
| White | 0.789 (0.787, 0.791) | 0.059 (0.053, 0.065) | 1.015 (1.011, 1.019) | 0.013 (0.012, 0.014) |
| Unknown | 0.826 (0.803, 0.850) | -0.049 (-0.259, 0.160) | 1.034 (0.920, 1.149) | 0.010 (0.002, 0.017) |
| **Hospital discharge diagnosis** |  |  |  |  |
| Injury (e.g., fractures) | 0.784 (0.780, 0.788) | 0.155 (0.127, 0.183) | 1.100 (1.081, 1.118) | 0.013 (0.011, 0.014) |
| Circulatory | 0.753 (0.749, 0.757) | -0.016 (-0.036, 0.003) | 0.984 (0.965, 1.004) | 0.013 (0.011, 0.015) |
| Musculoskeletal system | 0.866 (0.860, 0.871) | 0.566 (0.503, 0.629) | 1.271 (1.240, 1.302) | 0.016 (0.014, 0.017) |
| Neoplasms | 0.771 (0.763, 0.779) | -0.010 (-0.014, -0.006) | 0.893 (0.860, 0.925) | 0.018 (0.013, 0.023) |
| Other | 0.745 (0.742, 0.748) | -0.064 (-0.074, -0.054) | 0.938 (0.928, 0.947) | 0.010 (0.008, 0.011) |

Abbreviations: c-statistic, concordance statistic; CI, confidence interval; ICI, integrated calibration index, Eavg, the average absolute difference between observed and predicted probabilities over the range of predicted probabilities

a Racial and ethnic categories were based on the Medicare Research Triangle Institute race code in the Medicare Master Beneficiary Summary File. Race and ethnicity was not included in the model.

**Supplementary Table S13:** Optimism-corrected performance of the model for the outcome of successful community discharge across subgroups by age, sex, race and ethnicity, and clinical diagnosis

| **Outcome: successful community discharge** | | | | |
| --- | --- | --- | --- | --- |
| **Subgroup** | **c-statistic (95% CI)** | **Calibration intercept (95% CI)** | **Calibration slope (95% CI)** | **ICI (Eavg) (95% CI)** |
| **Age group** |  |  |  |  |
| 66-74 | 0.754 (0.751, 0.757) | -0.018 (-0.026, -0.011) | 1.054 (1.040, 1.067) | 0.011 (0.010, 0.013) |
| 75-84 | 0.738 (0.736, 0.740) | -0.014 (-0.021, -0.008) | 1.034 (1.023, 1.045) | 0.011 (0.010, 0.013) |
| 85+ | 0.698 (0.696, 0.701) | 0.010 (0.006, 0.014) | 0.921 (0.910, 0.932) | 0.012 (0.011, 0.014) |
| **Sex** |  |  |  |  |
| Male | 0.723 (0.720, 0.725) | -0.001 (-0.003, 0.001) | 0.998 (0.988, 1.008) | 0.009 (0.007, 0.010) |
| Female | 0.732 (0.730, 0.733) | 0.000 (-0.002, 0.002) | 1.000 (0.994, 1.006) | 0.013 (0.012, 0.014) |
| **Race/ethnicity^a^** |  |  |  |  |
| Asian/Pacific Islander | 0.736 (0.724, 0.748) | 0.302 (0.251, 0.354) | 0.920 (0.863, 0.976) | 0.062 (0.051, 0.072) |
| Black | 0.738 (0.733, 0.743) | 0.143 (0.122, 0.164) | 0.949 (0.925, 0.974) | 0.031 (0.027, 0.035) |
| Hispanic | 0.724 (0.716, 0.732) | 0.252 (0.216, 0.288) | 0.884 (0.845, 0.924) | 0.053 (0.045, 0.060) |
| Native American | 0.715 (0.694, 0.736) | 0.074 (-0.023, 0.171) | 0.936 (0.825, 1.048) | 0.010 (-0.004, 0.023) |
| Other | 0.750 (0.728, 0.772) | 0.224 (0.136, 0.312) | 0.999 (0.888, 1.110) | 0.045 (0.029, 0.061) |
| White | 0.729 (0.727, 0.731) | -0.045 (-0.049, -0.042) | 1.034 (1.030, 1.038) | 0.013 (0.012, 0.014) |
| Unknown | 0.751 (0.730, 0.771) | 0.069 (-0.035, 0.173) | 1.000 (0.893, 1.107) | 0.012 (0.001, 0.024) |
| **Hospital discharge diagnosis** |  |  |  |  |
| Injury (e.g., fractures) | 0.719 (0.715, 0.723) | -0.022 (-0.031, -0.013) | 1.044 (1.025, 1.064) | 0.011 (0.009, 0.013) |
| Circulatory | 0.705 (0.702, 0.708) | 0.001 (-0.001, 0.004) | 0.991 (0.972, 1.011) | 0.008 (0.005, 0.010) |
| Musculoskeletal system | 0.769 (0.764, 0.775) | -0.312 (-0.339, -0.286) | 1.277 (1.254, 1.300) | 0.031 (0.028, 0.033) |
| Neoplasms | 0.731 (0.722, 0.740) | 0.010 (-0.001, 0.020) | 1.065 (1.012, 1.117) | 0.012 (0.006, 0.018) |
| Other | 0.693 (0.690, 0.695) | 0.008 (0.007, 0.009) | 0.913 (0.904, 0.923) | 0.012 (0.010, 0.013) |

Abbreviations: c-statistic, concordance statistic; CI, confidence interval; ICI, integrated calibration index, Eavg, the average absolute difference between observed and predicted probabilities over the range of predicted probabilities

a Racial and ethnic categories were based on the Medicare Research Triangle Institute race code in the Medicare Master Beneficiary Summary File. Race and ethnicity was not included in the model.

**Supplementary References**

1. Vickers AJ, Calster BV, Steyerberg EW. Net benefit approaches to the evaluation of prediction models, molecular markers, and diagnostic tests. *BMJ*. 2016;352:i6. doi:10.1136/bmj.i6

2. Vickers AJ, van Calster B, Steyerberg EW. A simple, step-by-step guide to interpreting decision curve analysis. *Diagnostic and Prognostic Research*. 2019;3(1):18. doi:10.1186/s41512-019-0064-7

3. Vickers AJ, Elkin EB. Decision curve analysis: a novel method for evaluating prediction models. *Med Decis Making*. 2006;26(6):565-574. doi:10.1177/0272989X06295361

4. Van Calster B, Wynants L, Verbeek JFM, et al. Reporting and Interpreting Decision Curve Analysis: A Guide for Investigators. *European Urology*. 2018;74(6):796-804. doi:10.1016/j.eururo.2018.08.038

5. Harrell F. Statistically efficient ways to quantify added predictive value of new measurements. November 15, 2020. Accessed July 14, 2021. https://www.fharrell.com/post/addvalue/
